# Light-Triggered Switching of Metallosupramolecular Polymer Systems

**DOI:** 10.1021/acsmacrolett.5c00205

**Published:** 2025-05-20

**Authors:** Luca Bertossi, Marta Oggioni, Georges J. M. Formon, Christoph Weder

**Affiliations:** Adolphe Merkle Institute, Polymer Chemistry and Materials, 27211 University of Fribourg, Chemin des Verdiers 4, 1700 Fribourg, Switzerland

## Abstract

Metallosupramolecular polymers (MSPs) are formed through the formation of coordination complexes between monomers that contain multiple ligands and suitable metal salts. The assembly of MSPs is generally dynamic and reversible, which leads to stimuli-responsive materials and enables functions such as healing or recycling. Heat is arguably the most widely employed stimulus to manipulate MSPs, but the level of control that can be achieved is limited. Here, we report light-responsive MSP systems, whose response is based on an opto-chemical transduction principle. We combined the photoacid generator 2-(4-methoxy­styryl)-4,6-bis­(trichloromethyl)-1,3,5-triazine (MBTT) with poly­(acrylates) that comprise a few mol % of the 2,6-bis­(1′-methyl-benzimidazolyl)­pyridine (Mebip) ligand. The latter forms supramolecular cross-links upon the addition of metal salts, such as Zn^2+^, Eu^3+^, and Cu^2+^. We utilized titration experiments, optical spectroscopy, and rheology on model compounds and polymer systems to demonstrate that the MSP network can be rapidly disassembled upon optical activation of the photoacid generator, on account of protonation of the ligand and dissociation of the ML complex. Optorheological experiments reveal that the rheological properties of gels based on the MSP network, MBTT, and chlorobenzene can be drastically altered in an on-demand fashion by exposure to UV light.

## Graphical abstract

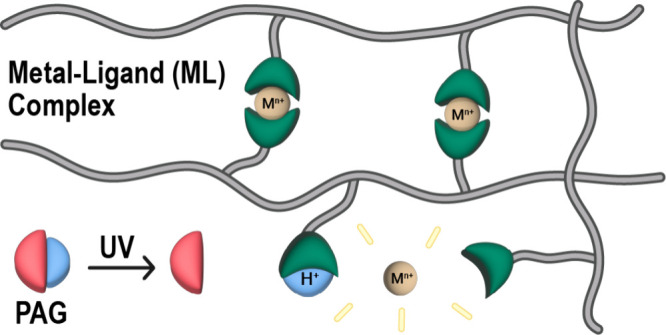

Stimuli-responsive polymers alter their properties in response to an external stimulus and offer a range of functions, including mechanical morphing and actuation, healability, and mechanochemical transduction.^1–5^ One widely employed approach to impart polymers with stimuli-responsive characteristics is the incorporation of supramolecular binding motifs whose assembly can be controlled by external cues such as chemicals, electromagnetic radiation, or heat.^6–12^ Metal–ligand complexes have attracted considerable interest for the design of stimuli-responsive metallosupramolecular polymers (MSPs), as the association and dynamicity of the interactions can be tuned by the nature of the metal salt and the ligand.^12–16^ Heat is arguably the most widely employed stimulus to manipulate MSPs in their solid state, for example, to achieve healing,^17–22^ debonding,^23^ or to program shape-memory polymers.^21,24^ Even though heat can be generated by the conversion of light, an oscillating magnetic field, or an electrical current, the level of control that can be achieved is limited if the underlying process driving the supramolecular (dis)­assembly is ultimately a temperature change.^8,25^ Intriguingly, examples of MSPs, and other supramolecular polymers, whose properties can be altered under isothermal conditions are rare.^26^ With the aim of advancing the specificity and functionality of adaptive MSPs, we explored a materials systems approach. We combined an MSP network based on metal–ligand (ML) complexes involving the widely employed 2,6-bis­(1′-methyl-benzimidazolyl)­pyridine (Mebip) ligand^17,27–29^ with a photoacid generator (PAG)^30,31^ that serves as an opto-chemical signal transducer (Figure 1). The PAG is activated by UV light and causes the disassembly of the MSP network by protonating the ligand, thereby changing the properties of the system. Our work complements recent reports on light-responsive hydrogels based on PAGs and low-molecular-weight gelators,^32^ sol–gel transitions achieved by photooxidative switching of polymer networks assembled with metal–organic cages,^33^ and the light-induced (dis)­assembly of organogels featuring Pd_n_L2_n_-type cross-links using a metastable photoacid.^34,35^

**Figure 1:**
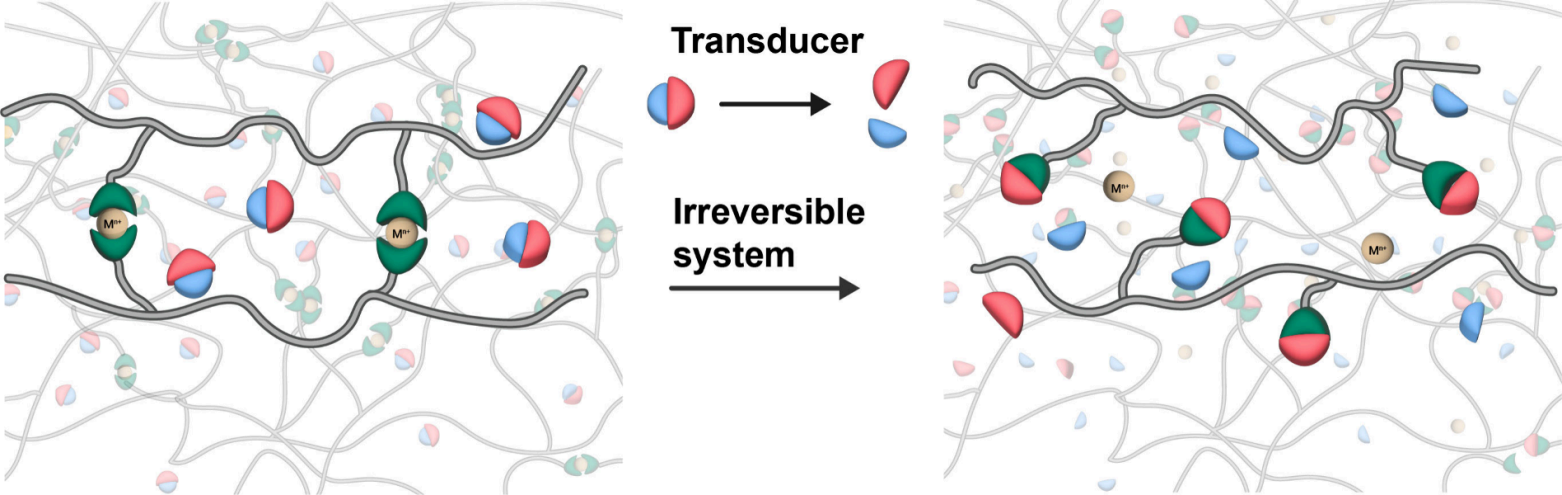
Schematic of the design and function of the metallosupramolecular polymer (MSP) systems investigated here. They consist of a photoacid generator (PAG) as a transducer and an MSP network. Upon light-triggered activation of the PAG and *in situ* acid production, the metal–ligand complexes that cross-link the polymer dissociate due to protonation of the ligand.

The design of the here-investigated systems was guided by our group’s previous experiences with different MSPs.^17,19,23,36,37^ To select a suitable ligand that forms robust ML complexes that can be dissociated by protonation, and to develop an understanding of the protonation process, we carried out titrations with low-molecular-weight model compounds of Mebip^27,28,38,39^ (ethylhexyl-Mebip, **EH-Mebip**) and its recently reported bidentate analog 6-(1′-methylbenzimidazolyl)-pyridine-3-ol (**EH-MBP**) (Figure 2a,c).^40^ We first titrated a solution of **EH-MBP** in acetonitrile (MeCN, *c* = 24 μM) with hydrochloric acid (HCl) and monitored the ligand protonation by UV–vis absorption spectroscopy (Figure 2a). The absorption spectra reveal the gradual increase of an absorption band that is initially centered around 313 nm (π → π* transition of the ligand) and shifts to 314 nm (π → π* transition of the protonated ligand) upon protonation.

**Figure 2:**
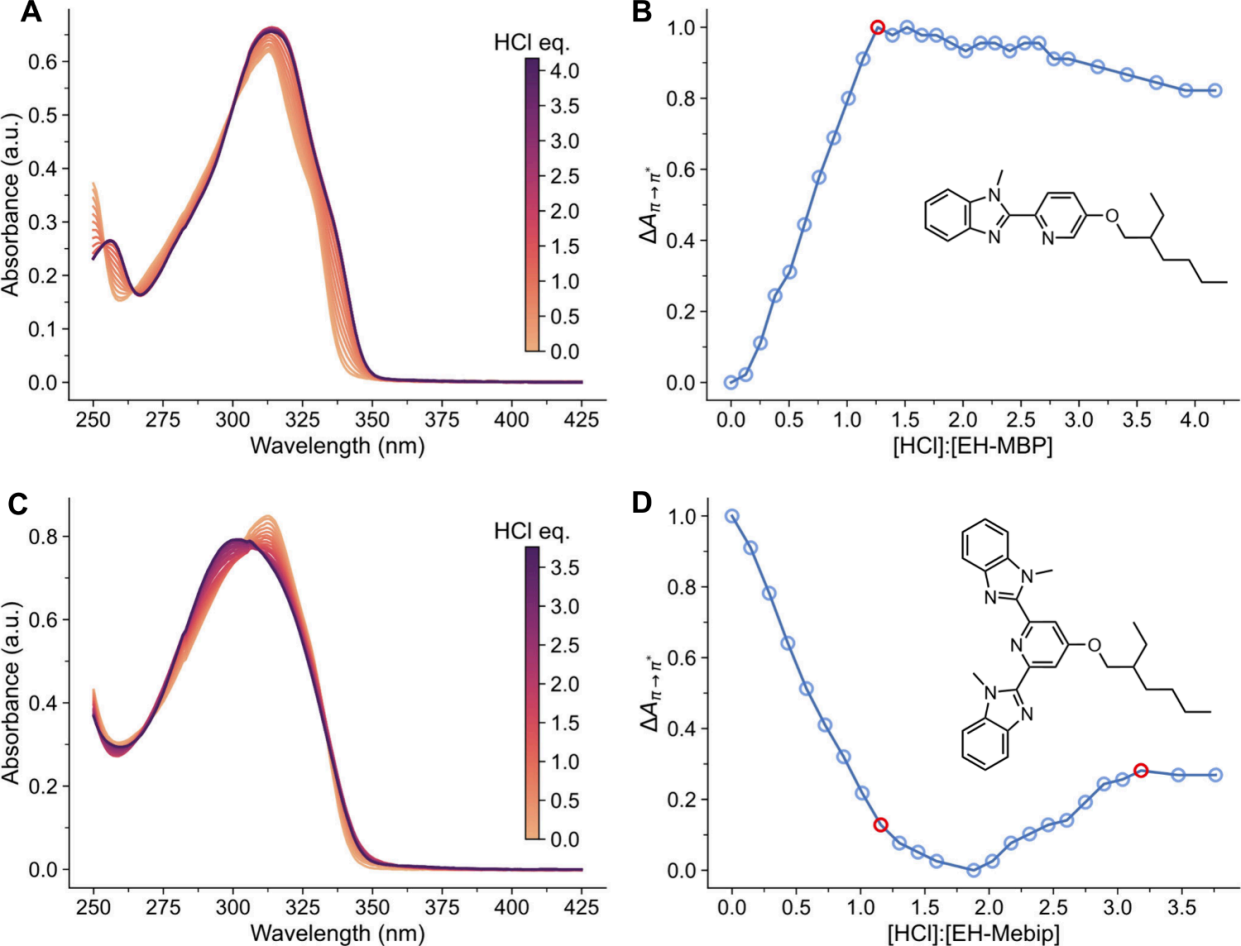
Titrations of solutions of the model ligands **EH-MBP** (A, B) and **EH-Mebip** (C, D) with HCl, monitored by UV–vis absorption spectroscopy. (A, C) Absorption spectra acquired upon the addition of aliquots of HCl (*c* = 3 mM) to solutions of (A) **EH-MBP** (*c* = 24 μM) and (C) **EH-Mebip** (*c* = 21 μM). (B, D) Plots of the normalized change of the absorbance at the peak maximum against the (B) [HCl]:[**EH-MBP**] and (D) [HCl]:[**EH-Mebip**] ratio. The end points, shown in red, mark the ends of regimes in which the respective ligand is singly or doubly protonated. For all solutions, MeCN served as the solvent.

A plot of the change in absorbance at the maximum of this band against the [HCl]:[**EH-MBP**] ratio reveals an end point at an [HCl]:[**EH-MBP**] ratio of ca. 1.3,^41^ indicating that this bidentate ligand can be protonated only once at reasonable acid concentrations (Figure 2b). This observation is consistent with studies of bipyridines, for which single protonation was reported.^42^ The UV–vis absorption spectra recorded when **EH-Mebip** was titrated with HCl in the same manner are shown in Figure 2c. In this case, the absorption band initially centered at 313 nm (π → π* transition of the free ligand) displays a hypsochromic shift to 300 nm (π → π* transition of the protonated ligand) upon HCl addition. A plot of the change in absorbance (again measured at the shifting wavelength that marks the band’s peak) against the [HCl]:[**EH-Mebip**] ratio is shown in Figure 2d. Applying a previously reported methodology,^41^ this plot reveals two inflection points and two end points (roots of the second and first derivatives respectively), from this we discern end points at [HCl]:[**EH-Mebip**] ratios of ca. 1.2 and 3.2 where the overlap of bands and the result of EH-MBP are considered (Figure 2d, Figure S1, additional discussion in the Supporting Information), thus indicating that **Mebip** can be protonated twice; also this finding agrees with reports on the protonation of other tridentate ligands.^41,42^ The UV–vis titration data are corroborated by ^1^H NMR experiments in which we monitored the protonation of **EH-Mebip** with trifluoroacetic acid (TFA). The NMR spectra reveal pronounced upfield shifts of the resonances of diagnostic protons upon TFA addition (Figure S2a,b). A plot of the change in the chemical shift against the [TFA]:[**EH-Mebip**] ratios shows again two end points at values of ca. 1.5 and 5.5 (Figure S2c). Thus, despite two or three available protonation sites, these ligands can only be protonated once (**EH-MBP**) or twice (**EH-Mebip**). Since **EH-Mebip** displays a higher binding constant with transition metals than **EH-MBP**,^27,40^ we selected the former for further experiments, expecting that it would afford systems with a more robust “on” state than **EH-MBP**.

We next investigated if Mebip complexes with Zn^2+^ salts, which our group and others exploited previously to construct different types of MSPs,^17–19,21,23,27,38,39^ dissociate in acidic conditions. This was accomplished by titrating MeCN solutions of the complex made from Zn­(OTf)_2_ and **EH-Mebip** (**Zn­(EH-Mebip)**_**2**_) with strong acids. Figure 3a shows the UV–vis absorption spectra acquired for the titration of **Zn­(EH-Mebip)**_**2**_ with HCl. Gratifyingly, the metal-to-ligand charge transfer (MLCT) band associated with the complex at 335 nm decreases upon acid addition, while the π→π* band of the protonated **EH-Mebip** ligand at 313 nm develops concomitantly. A plot of the change in the ratio of the absorbances at these wavelengths against the [HCl]:[**Zn­(EH-Mebip)**_**2**_] ratio shows that complete decomplexation of **Zn­(EH-Mebip)**_**2**_ is achieved at a ratio of ca. 4:1 (Figure 3c). Similar titrations with TFA and trifluoromethanesulfonic acid (TFSA) show that TFA does not dissociate the complex while TFSA does, indicating that a strong acid is required. Since HCl also introduces chloride ions that can coordinate with Zn^2+^ and might serve as a competitive binder, we also titrated **Zn­(EH-Mebip)**_**2**_ with tetraethylammonium chloride (TEAC, Figure 3b,c). The results show that chloride ions indeed dissociate the complex, which suggests that the HCl-induced decomplexation of **Zn­(EH-Mebip)**_**2**_ is due to a combination of **EH-Mebip** protonation and coordination of Cl^–^ and Zn^2+^. Finally, we explored if **EH-Mebip** complexes with other metal salts can be dissociated in a similar manner. We selected Eu­(OTf)_3_, a lanthanide salt known to form more dynamic 1:3 ML complexes with **Mebip**, and Cu­(OTf)_2_, which Mebip can bind in 1:1 and 1:2 geometries.^43,44^ We confirmed these complexation behaviors with titrations of **EH-Mebip** with Cu­(OTf)_2_ and Eu­(OTf)_3_ in MeCN; as expected based on previous works,^45,46^ these experiments reveal the formation of **Cu­(EH-Mebip)**_**2**_, **Cu­(EH-Mebip)**, **Eu­(EH-Mebip)**_**3**_, and **Eu­(EH-Mebip)**_**2**_ (Figure S4). We titrated MeCN solutions of these complexes with HCl and monitored the effect again with UV–vis absorption spectroscopy (Figure 3d). The data show that the ease of dissociation by protonation decreases in the order **Eu­(EH-Mebip)**_**2**_ > **Zn­(EH-Mebip)**_**2**_ > **Eu­(EH-Mebip)**_**3**_ > **Cu­(EH-Mebip**), while **Cu­(EH-Mebip)**_**2**_ complexes hardly dissociated, even at a 10-fold excess of HCl. The results are consistent with the Irving-Williams series^47^ and reflect that the response of metal-Mebip complexes to a strong acid can be tailored over a wide range. **Eu­(EH-Mebip)**_**2**_ exhibits the highest dissociation degree upon acid addition. However, previous reports indicate that Eu-based materials tend to display poorer mechanical properties due to their low binding equilibrium constant, when compared to Zn-Mebip complexes.^17–19,23,38^ Thus, **Zn­(EH-Mebip)**_**2**_ was selected for the systems discussed below, as it is known to yield materials with robust mechanical properties, combined with relatively high acid-lability.

**Figure 3:**
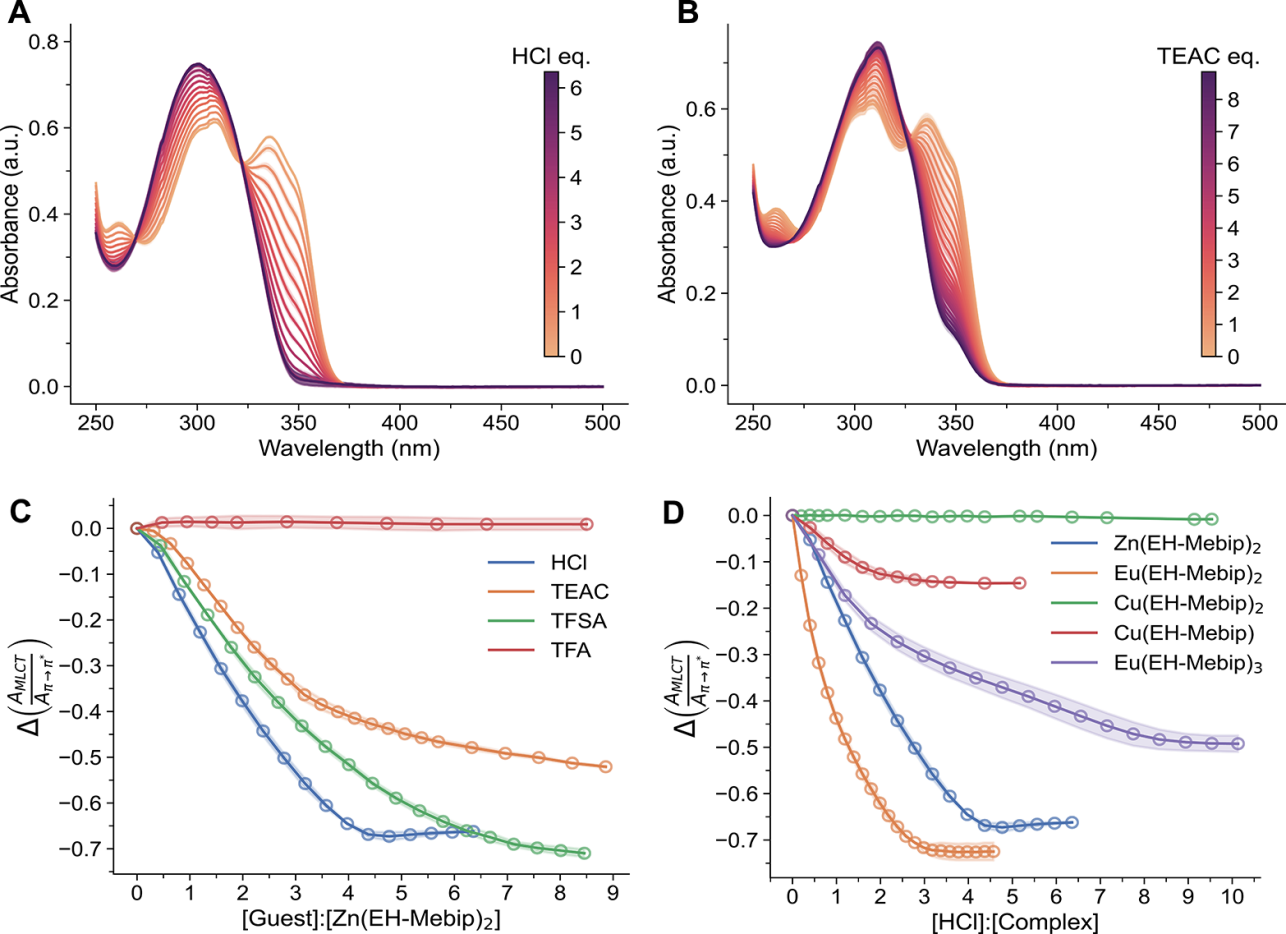
Titrations of solutions of the model complexes with different acids and TEAC, monitored by UV–vis absorption spectroscopy. (A) Absorption spectra acquired upon the addition of aliquots of HCl (*c* = 1.65 mM) and (B) TEAC (*c* = 1.31 mM) to solutions of **Zn­(EH-Mebip)**_**2**_ (*c* = 11.5 μM). (C) Titrations of **Zn­(EH-Mebip)**_**2**_ (*c* = 11.5 μM) with HCl (*c* = 1.65 mM), TFSA (*c* = 1.85 mM), TFA (*c* = 3.25 mM), or TEAC (*c* = 1.31 mM). The plot shows the ratio of the absorbances at 335 nm (MLCT band of **Zn­(EH-Mebip)**_**2**_) and ca. 313 nm (π → π* band of the protonated ligand, the absorbance was recorded at the peak maximum) against the [guest]:[**Zn­(EH-Mebip)**_**2**_] ratio (data taken from Figures 3a,b and S3). (D) Titrations of **M­(EH-Mebip)**_*x*_ complexes (M = Zn^2+^, Eu^3+^, and Cu^2+^, *c* = 11.5 μM) with HCl (*c* = 1.65 mM). The plot shows the ratio of the absorbances at the MLCT peak (Figure S5) and ca. 313 nm (π → π* band of the protonated ligand, the absorbance was recorded at the peak maximum) against the [HCl]:[**M­(EH-Mebip)**_*x*_] ratio. All scatter points are the mean of at least two repeats and a nonparametric 95% confidence interval is indicated by the shading. All solutions are in MeCN.

Based on the above-discussed model studies, we elected to utilize 2-(4-methoxystyryl)-4,6-bis­(trichloromethyl)-1,3,5-triazine (MBTT), a well-known photoacid generator that generates HCl upon UV irradiation, as the PAG for the envisioned systems (Figure 4b).^48,49^ The possibility of decomplexing **Zn­(EH-Mebip)**_**2**_ upon activation of MBTT with light was demonstrated by irradiating MeCN solutions of the complex (*c* = 11.5 μM) and MBTT with [MBTT]:[**Zn­(EH-Mebip)**_**2**_] ratios of 0.17, 1, and 5 using a focused UV LED (λ = 365 nm, *P* = 1.29 W, power density on the sample ca. 190 mW/cm^2^) and monitoring the changes by UV–vis absorption spectroscopy. Note that the absorption spectra were corrected for changes associated with the PAG dissociation (Figure S7) and small baseline offsets arise from imperfect correction (Figures 4 and S6). A comparison of the spectra thus recorded and the spectra collected upon titration of **Zn­(EH-Mebip)**_**2**_ with HCl (Figure 3a) clearly demonstrates that the optical activation of MBTT triggers the decomplexation. Figure 4a, which shows the absorption spectra for an [MBTT]:[**Zn­(EH-Mebip)**_**2**_] ratio of 1, indicates that 1 equiv of MBTT dissociates the complex to the same extent as ca. 2 equiv of HCl when irradiated for up to 100 min (Figure S8), which is consistent with the reported release of more than one proton per MBTT.^49^ Indeed, titrations, in which Rhodamine B was used as an acid indicator, show under the conditions used here, MBTT releases ca. 1 equiv of HCl within the first 5 min of irradiation, after which the release slows down such that irradiation of ca. 100 min is required to release another 1 equiv of HCl (Figure S9). This is further supported by mass spectrometry experiments, which reveal the loss of two chlorine atoms for each MBTT after prolonged UV exposure (Figure S10). Thus, less MBTT is required to dissociate **Zn­(EH-Mebip)**_**2**_ if UV is applied over longer periods of time at a high intensity. Conversely, if an excess of MBTT is used (e.g., 5 equiv, cf. Figure 4b), rapid (5 min under the conditions employed) and full decomplexation can be achieved. A comparison of Figure 4b and a plot that shows the optical changes of a reference solution containing MBTT only, demonstrates that the rate-determining step is the decomposition of the PAG (Figure S7), which in turn is limited by the power density applied (Figure S11). These results are corroborated by a ^1^H NMR spectroscopic analysis of the process (Figure S12), which reveals that full dissociation is only achieved after several hours with a low power density applied. The slow kinetics are also related to the high concentration of MBTT and the high optical density that it imparts. The result brings out one critical limitation of the approach, i.e., that a low optical density is required to achieve fast switching. As it is known that chloroform can degrade in the presence of UV light into phosgene and HCl, we carried out a control experiment that involved irradiating a CDCl_3_ solution of only **Zn­(EH-Mebip)**_**2**_ under identical conditions; gratifyingly, in the absence of MBTT, the complex is unaffected (Figure S13).

**Figure 4:**
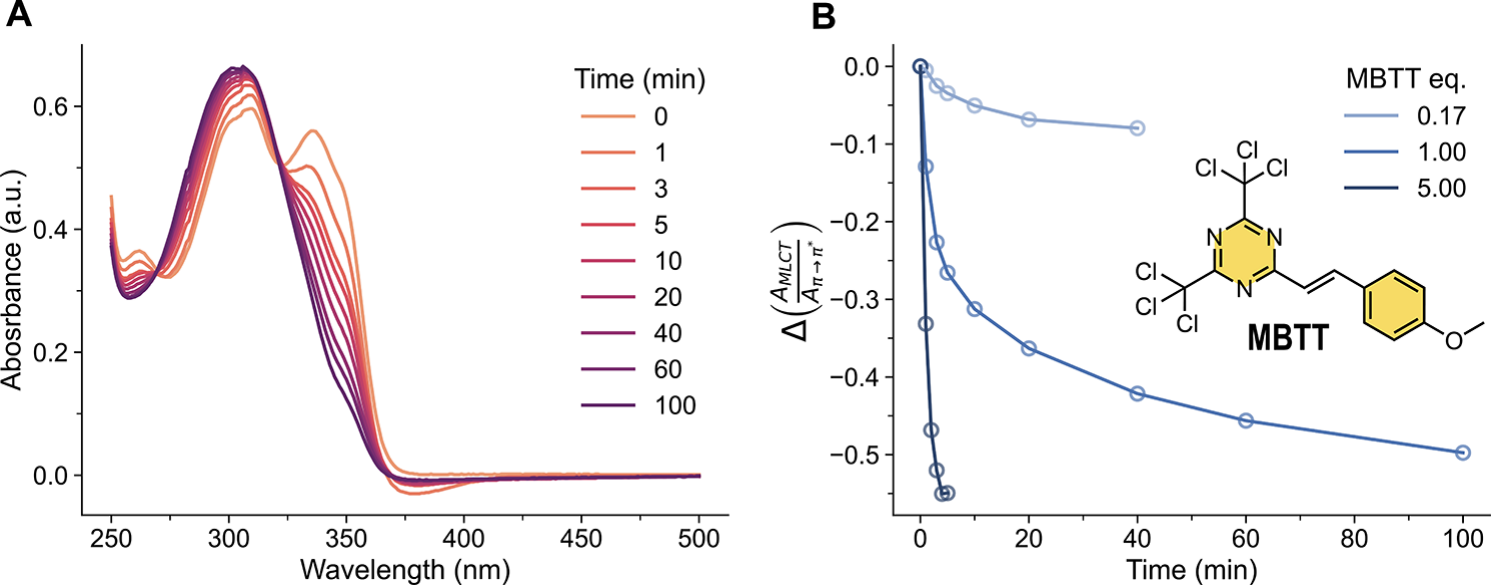
Decomplexation of **Zn­(EH-Mebip)**_**2**_ through the optical activation of MBTT. (A) A solution of **Zn­(EH-Mebip)**_**2**_ (*c* = 11.5 μM) and MBTT (1 equiv) in MeCN was irradiated with UV light (λ = 365 nm, *P* = 1.29 W, power density on the sample ca. 190 mW/cm^2^) and the resulting spectral changes were monitored by UV–vis absorption spectroscopy as a function of irradiation time. (B) The experiment was repeated, but the [MBTT]:[**Zn­(EH-Mebip)**_**2**_] ratio was varied to assume values of 0.17, 1.0, and 5.0. The figure shows a plot of the ratio of the absorbances at 335 nm (MLCT band of **Zn­(EH-Mebip)**_**2**_) and ca. 313 nm (π → π* band of the protonated ligand, the absorbance was recorded at the peak maximum) as a function of irradiation time. The chemical structure of MBTT is also depicted.

We based the MSP systems on statistical copolymers of *n*-butyl acrylate (**BA**), a rubbery, amorphous polymer with subambient glass-transition temperature (*T*_g_), and a **Mebip-acrylate** (**MBA**), which was prepared by adapting a reported protocol (Figure 5a).^36^ Statistical copolymers of **BA** and **MBA** (poly­(*n*-butyl acrylate-*co*-Mebip acrylate) were prepared by reversible addition–fragmentation chain transfer (RAFT) polymerization (Scheme S1) using an adapted literature method.^21,36,50^ We systematically varied the number-average molecular weight (*M*_n_) in the range of 10000–50000 g/mol and the mole fraction of the **MBA** between 4 and 10 mol % (Table S1). These materials are referred to as **PBA**-*co* **-MBA**_*xx*_ **-YY**, where XX indicates the mole fraction of **MBA** in the polymer and YY its *M*_n_ in kg/mol. ^1^H NMR spectra show that the mole fraction of **MBA** residues in the polymer generally corresponds to that in the feed, diffusion-ordered NMR spectra reveal that **MBA** is indeed incorporated into the polymer backbone, and size-exclusion chromatography shows that the dispersity *Đ* is low (1.1–1.3), except for the highest concentration of **MBA** (*Đ* = 1.7) (Table S1, Scheme S1, SI page S29).

**Figure 5:**
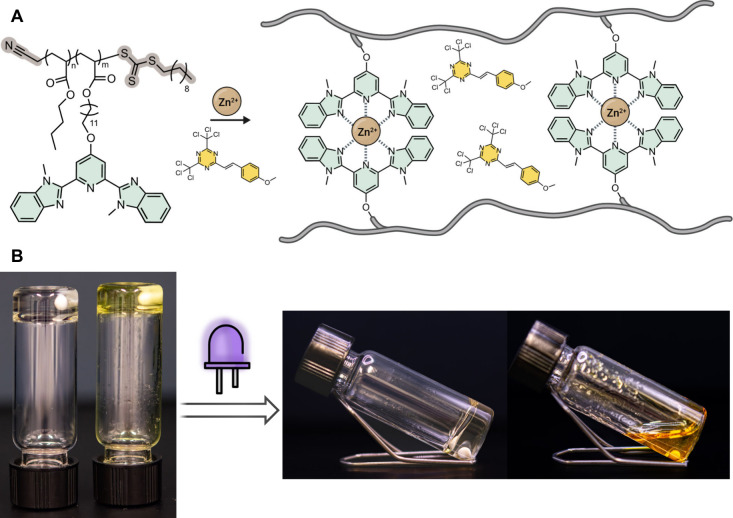
MSP gels were prepared using the copolymers of **PBA**-*co* **-MBA**_*xx*_ **-YY** following the addition of Zn­(OTf)_2_. (A) Chemical structure of **PBA**-*co* **-MBA**_*xx*_ **-YY** and an illustration of the components in **Zn­(PBA**-*co* **-MBA**_**5**_ **-43)/MBTT** gels. (B) Pictures of as-prepared **Zn­(PBA**-*co* **-MBA**_**5**_ **-43)** (colorless) and **Zn­(PBA**-*co* **-MBA**_**5**_ **-43)/MBTT** (yellow) gels in chlorobenzene (left) and the solution obtained after irradiating the gels for 10 min with UV light (365 nm, 90 mW/cm^2^). Both gels contain 12 wt % of the MSP and the MBTT gel additionally 1.2 wt % of MBTT ([MBTT]:[**Zn­(MBA)**_**2**_] = 1.5).

MSP formation was achieved by adding stoichiometric amounts of Zn­(OTf)_2_ dissolved in MeOH/MeCN (1:9) to dilute solutions (*c* = 0.0167 g/mL) of **PBA**-*co* **-MBA**_*xx*_ **-YY** in CHCl_3_, and complex formation was again monitored by UV/vis absorption spectroscopy (Figure S14). Once complete coordination in a 2:1 ratio of Mebip to Zn­(OTf)_2_ was confirmed, solutions of **Zn­(PBA**-*co* **-MBA**_*xx*_ **-YY)** were slowly air-dried and subsequently reswelled with a solvent of choice to form the MSP organogels. Table S1 shows that **Zn­(PBA**-*co* **-MBA**_**6**_ **-13)**, i.e., the MSP with the lowest *M*_n_ and a rather low cross-link density, dissolves, rather than gels in CHCl_3_, whereas **Zn­(PBA**-*co* **-MBA**_**10**_ **-29)**, which has the highest cross-link density of all materials studied, hardly swells in CHCl_3_. The MSP with intermediate cross-link density and *M*_n_, **Zn­(PBA**-*co* **-MBA**_**5**_ **-43)**, forms a robust gel with CHCl_3_.

To identify a high-boiling solvent suited for swelling the MSPs and performing rheological measurements, screening experiments with **Zn­(PBA**-*co* **-MBA**_**5**_ **-43)** were performed (). The data shows that chlorinated solvents swell **Zn­(PBA**-*co* **-MBA**_**5**_ **-43)** the best and that self-supporting gels with a solvent mass fraction of >90% can be made. We thus carried out all subsequent experiments with gels containing 12 wt % **Zn­(PBA**-*co* **-MBA**_**5**_ **-43)** relative to the solvent. Corresponding gels that additionally contain MBTT (unless otherwise noted, based on **Zn­(PBA**-*co* **-MBA**_**5**_ **-43)** and 1.2 wt % MBTT relative to the solvent, in which case [MBTT]:[**Zn­(MBA)**_**2**_] = 1.5) were made in a similar manner by adding the MBTT to the MSP solution before drying.

A simple gel inversion test of the **Zn­(PBA**-*co* **-MBA**_**5**_ **-43)/MBTT** chlorobenzene gel shows that a light-triggered gel-to-sol transition is achieved when the gel is irradiated for 10 min with low-intensity UV light (365 nm, incident power = 90 mW/cm^2^), whereas gels without MBTT remain unaffected (Figures 5 and S16). A 10-fold reduction of the MBTT concentration (0.12 wt %, [MBTT]:[**Zn­(MBA)**_**2**_] = 0.15) affords gels that soften upon UV irradiation under identical conditions, but even upon extending the irradiation time, no sol formation is observed (Figure S17). Thus, these qualitative experiments already reflect that the systems approach pursued here allows for the optical switching of mechanical properties, and that control is possible via the UV dose, as well as the concentration of the MBTT transducer in the gel.

Oscillatory shear rheology experiments were carried out to characterize the viscoelastic mechanical properties of **Zn­(PBA**-*co* **-MBA**_**5**_ **-43)** and **Zn­(PBA**-*co* **-MBA**_**5**_ **-43)/MBTT** gels in chlorobenzene. Both frequency sweep and amplitude sweep experiments were carried out. The difference between the two gels is negligible (Figure S18), indicating that the unactivated MBTT does not affect the rheological properties of the MSP gel. The frequency sweep data of both gels, recorded at a fixed strain γ = 1%, show a storage modulus *G*′ of 4 kPa at higher angular frequencies (ω), with an onset of softening at ω = 10 rad/s (). A crossover point between *G*′ and the loss modulus (*G*′′) traces is observed at ω_cross_ = 1 rad/s, where most of the elasticity is lost and the gel begins to flow. The amplitude sweeps of the two gels, show a transition from the linear viscoelastic region (LVE) into the nonlinear regime at a critical strain γ_crit_ = 36%, indicating that a fixed strain of γ = 1% at ω = 10 rad/s is well within the LVE. Hence, these parameters were used for subsequent *in situ* irradiation experiments. Self-healing is a characteristic of MSPs that has been explored in gels as well as in the solid state.^19,51^ We investigated this feature on the MBTT-free **Zn­(PBA**-*co* **-MBA**_**5**_ **-43)** gel by applying a constant frequency of ω = 10 rad/s and cycling between applied strains of 1 and 150%, i.e., well below and above γ_crit_ (). The data shows that **Zn­(PBA**-*co* **-MBA**_**5**_ **-43)** indeed exhibits self-healing behavior; we relate the fact that *G*′ and *G*′′ did not fully recover primarily to experimental artifacts, i.e., sample loss from between the rheometer plates (Supporting Movie M1).

The possibility of altering the mechanical properties of the metallosupramolecular gel systems upon exposure to UV light was quantitatively assessed by *in situ* UV rheological experiments. Thus, the **Zn­(PBA**-*co* **-MBA**_**5**_ **-43)** control and the **Zn­(PBA**-*co* **-MBA**_**5**_ **-43)/MBTT** gels in chlorobenzene ([MBTT]:[**Zn­(MBA)**_**2**_] = 1.5) were irradiated with UV light (385 nm, incident power = 146 mW/cm^2^) while carrying out time sweeps and measuring *G*′ and *G*′′ at γ = 1% and ω = 10 rad/s (Figure S18). Gratifyingly, in the case of the MBTT-containing gel, *G*′ and *G*′′ drop rapidly as soon as the UV light is turned on. The two traces intersect after approximately 30s of irradiation, which marks the crossover from a gel to a viscous liquid (Figure 6a, Figure S20); the moduli are further reduced upon continued UV exposure, but in comparison to the initial decrease, the effect is quite small. Radical species generated by MBTT are rapidly quenched by the excess solvent and inhibit side reactions involving the polymer, which would result in cross-linking; instead, here we observe softening.^49^ By contrast, the moduli of the MBTT-free reference gel remain largely unaffected by the UV light. The minor and much slower changes of *G*′ and *G*′′ that are observed in this case (Figure S20) may be related to light–heat conversion after light absorption by the metal–ligand complexes and a minor temperature increase that this process may cause.

**Figure 6:**
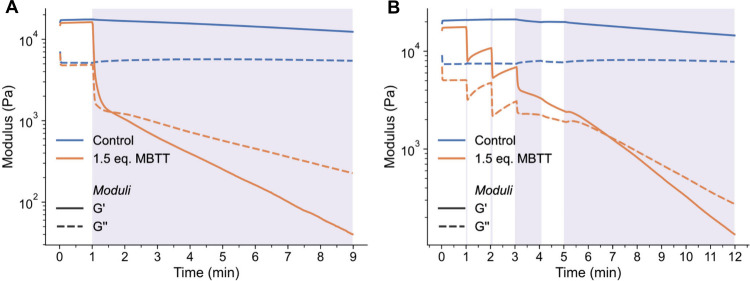
Optorheological experiments (time sweeps) of **Zn­(PBA**-*co* **-MBA**_**5**_ **-43)** and **Zn­(PBA**-*co* **-MBA**_**5**_ **-43)/MBTT** gels in chlorobenzene. (A) After an idle period of 1 min, the UV light was switched on (purple shade). (B) After an idle period of 1 min, the UV light was switched on for periods of 1 s, 1 s, 1 min, and 7 min (purple shades). Both gels contain 12 wt % of the MSP and the MBTT gel additionally 1.2 wt % of MBTT ([MBTT]:[**Zn­(MBA)**_**2**_] = 1.5). The experiments were conducted under nominally isothermal conditions at 25 °C with γ = 1%, ω = 10 rad/s, λ = 385 nm, and a power density on the samples of ca. 146 mW/cm^2^.

Surprised by the extremely fast softening reported in Figure 6a, we explored the rheological behavior upon irradiating the gels for shorter time intervals or with short pulses of light (Figures 6b, S21, and S22). While the MBTT-free gel remains again largely unaffected, immediate changes in both *G*′ and *G*′′ are observed for the **Zn­(PBA**-*co* **-MBA**_**5**_ **-43)/MBTT** gel, as reflected most strikingly by a plot of a rolling standard deviation of a 2 s window size on the measured moduli (Figure S22). Figure 6b reveals that even light pulses as short as 1 s are sufficient to elicit a pronounced reduction in *G*′ and *G*′′. Intriguingly, the moduli not only stop dropping after each light pulse, but they partially recover, although the magnitude of the recovery decreases with the irradiation dose. This behavior is consistent with acid concentration gradients in the sample, which are caused by the high optical density that the MBTT imparts. Since the light is introduced through the quartz bottom plate of the rheometer, the MBTT activation proceeds from the surface, and the gel near the plate liquefies more rapidly than the bulk. Figure 6b suggests that such spatial inhomogeneities can be limited by applying short light bursts and providing sufficient time for the acid to diffuse. Notwithstanding this effect, the mechanical changes displayed upon irradiating the **Zn­(PBA**-*co* **-MBA**_**5**_ **-43)/MBTT** gel with UV light are rapid and as pronounced as suggested by the gel inversion test discussed above.

In summary, acid titrations of bidentate and tridentate (1′-methyl-benzimidazolyl)­pyridine ligands and model complexes of these ligands and different metal salts, monitored by UV–vis absorption and ^1^H NMR spectroscopy, confirm the suitability of these building blocks to be used in metallosupramolecular polymer systems in which the photoacid generator MBTT serves as an opto-chemical signal transducer. Copolymers of butyl acrylate and 5 mol % of the tridentate Mebip ligand form self-supporting organogels upon the addition of Zn­(OTf)_2_, on account of the reversible cross-links formed by the metal–ligand complexes. Optorheological experiments reveal that the rheological properties of gels based on the MSP network, MBTT, and chlorobenzene can be drastically altered in an on-demand fashion by exposure to UV light. While the experiments show that the high optical density that the PAG imparts can lead to temporary spatial inhomogeneities, these rapidly disappear in the here-investigated gels on account of efficient acid diffusion. On the other hand, one may speculate that this effect can be exploited to trigger adhesive failure and thus achieve rapid debonding-on-demand in solid adhesives based on this approach.

## Supplementary Material





## Data Availability

Code written for this publication can be found at 10.5281/zenodo.15044644 or https://github.com/lucaAyt/titration_bot. The raw data is available for download at https://doi.org/10.5281/zenodo.15112907.

## Notes

Experimental procedures and characterization data including ^1^H NMR spectra, ^13^C NMR, and DOSY. Additional UV–vis and rheological data, images of samples, synthetic schemes, and tables summarizing the properties of polymers prepared can be found­(PDF)
Supporting Movie M1 (MP4)
