# Supplementary material for: Light-Triggered Switching of Metallosupramolecular Polymer Systems
Source: ACS Macro Lett. 2025 May 20;14(6):765–72. doi: 10.1021/acsmacrolett.5c00205 (PMC12177937; doi:10.1021/acsmacrolett.5c00205)
Supplement: Supplementary file 1 [file mz5c00205_si_001.pdf]

Light-triggered switching of metallosupramolecular polymer systems

Electronic Supplementary Information

*Luca Bertossi, Marta Oggioni, Georges J. M. Formon, Christoph Weder**

Adolphe Merkle Institute, Polymer Chemistry and Materials, University of Fribourg, Chemin des Verdiers 4,1700 Fribourg, Switzerland

Table of Contents

Supplementary Figures S1-S15 and Table S1 1

Model ligand titrations (S1) 1

Titrations of the model complex with different guests (S2) 3

Metal complexing data and HCl-induced dissociation (S4 – S5) 4

Model studies with MBTT (S6 – S13) 6

MSP investigations (Scheme S1, Table S1, S14 – S22) 11

Materials and Instrumentation 16

Synthetic Methods and Characterization 18

NMR Characterization 23

References 30

Supplementary Figures S1-S15 and Table S1

Model ligand titrations (S1)

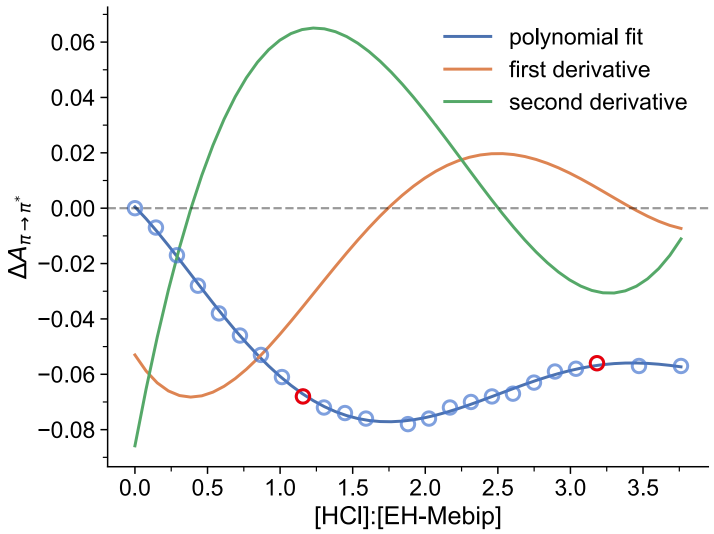

Figure S1. Further analysis of the data obtained from the titration of **EH-Mebip** and HCl monitored by UV-Vis spectroscopy. Hollow points indicate the original data, with the red dots indicating end points discerned after analysis. The data was fitted with a 5th-order polynomial with no physical significance. The first and second derivatives of the fit were calculated analytically and plotted. Two turning points and roots to the first and second derivative, respectively, indicate that there are only two regimes, and three species present throughout the titration (**EH-Mebip**, singly protonated and doubly protonated **EH-Mebip**). The roots of the second derivative indicate the pKa's, and the roots of the first indicate the endpoints. With prior knowledge of the **EH-MBP** endpoint and the low resolution between bands associated with the singly and doubly protonated **EH-Mebip** species (ca. 1.0 – 2.0), we discern that the first endpoint is at a lower ratio than provided by the first derivatives and is highlighted in red.

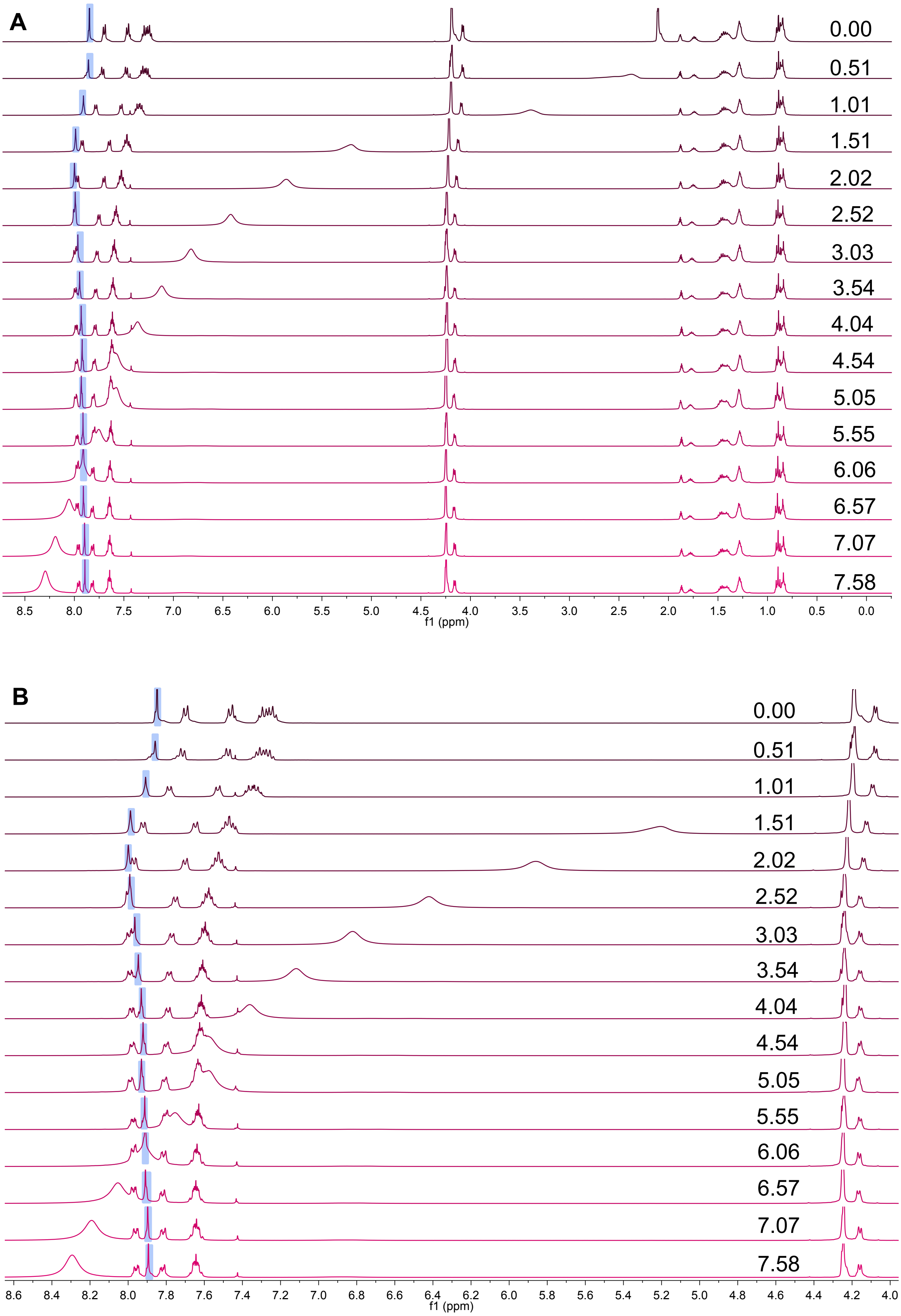
A
0.00
0.51
1.01
1.51
2.02
2.52
3.03
3.54
4.04
4.54
5.05
5.55
6.06
6.57
7.07
7.58
8.5 8.0 7.5 7.0 6.5 6.0 5.5 5.0 4.5 4.0 3.5 3.0 2.5 2.0 1.5 1.0 0.5 0.0
f1 (ppm)
B
0.00
0.51
1.01
1.51
2.02
2.52
3.03
3.54
4.04
4.54
5.05
5.55
6.06
6.57
7.07
7.58
8.6 8.4 8.2 8.0 7.8 7.6 7.4 7.2 7.0 6.8 6.6 6.4 6.2 6.0 5.8 5.6 5.4 5.2 5.0 4.8 4.6 4.4 4.2 4.0
f1 (ppm)

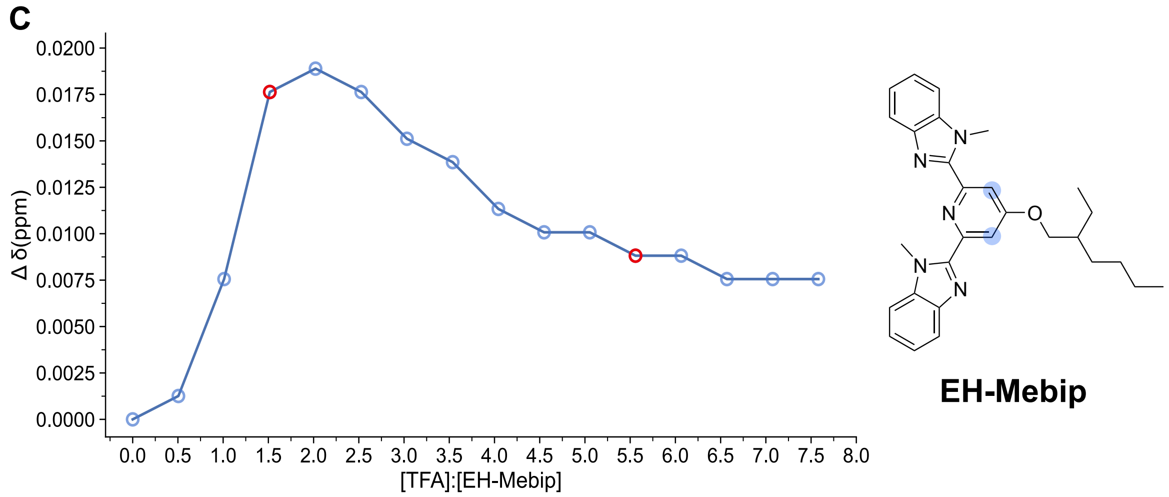

Figure S2. Titration of a solution of the model ligand **EH-Mebip** (c=38.8 mM in CD_3CN) with TFA (c=3.9 M in CD_3CN) using ^{1}H-NMR spectroscopy. (A,B) Stacks of ^{1}H-NMR spectra recorded for different [TFA]:[**EH-Mebip**] ratios, as annotated on the right side. The peak most influenced by the protonation of **EH-Mebip** is highlighted. It corresponds to the protons in the *meta* position to the nitrogen atom of the pyridine ring and is assigned in the chemical structure above by color code. (C) Plot of the chemical shift of the protons highlighted in the spectra against the [TFA]:[**EH-Mebip**] ratio (data extracted from A,B).

Titrations of the model complex with different guests (S2)

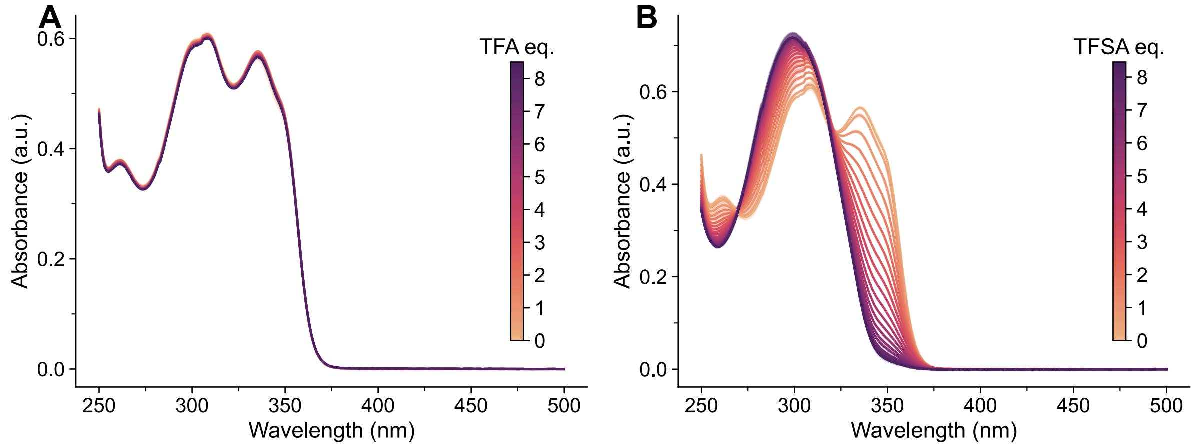

Figure S3. Titrations of model complexes with TFA and TFSA, monitored by UV-Vis absorption spectroscopy. The plots show absorption spectra acquired upon the addition of aliquots of (A) TFA (c=3.25 mM) and (B) TFSA (c=1.85 mM) to solutions of **Zn(EH-Mebip)$_2$** (c=11.5 μM). All solutions are based on MeCN.

Metal complexing data and HCl-induced dissociation (S4 – S5)

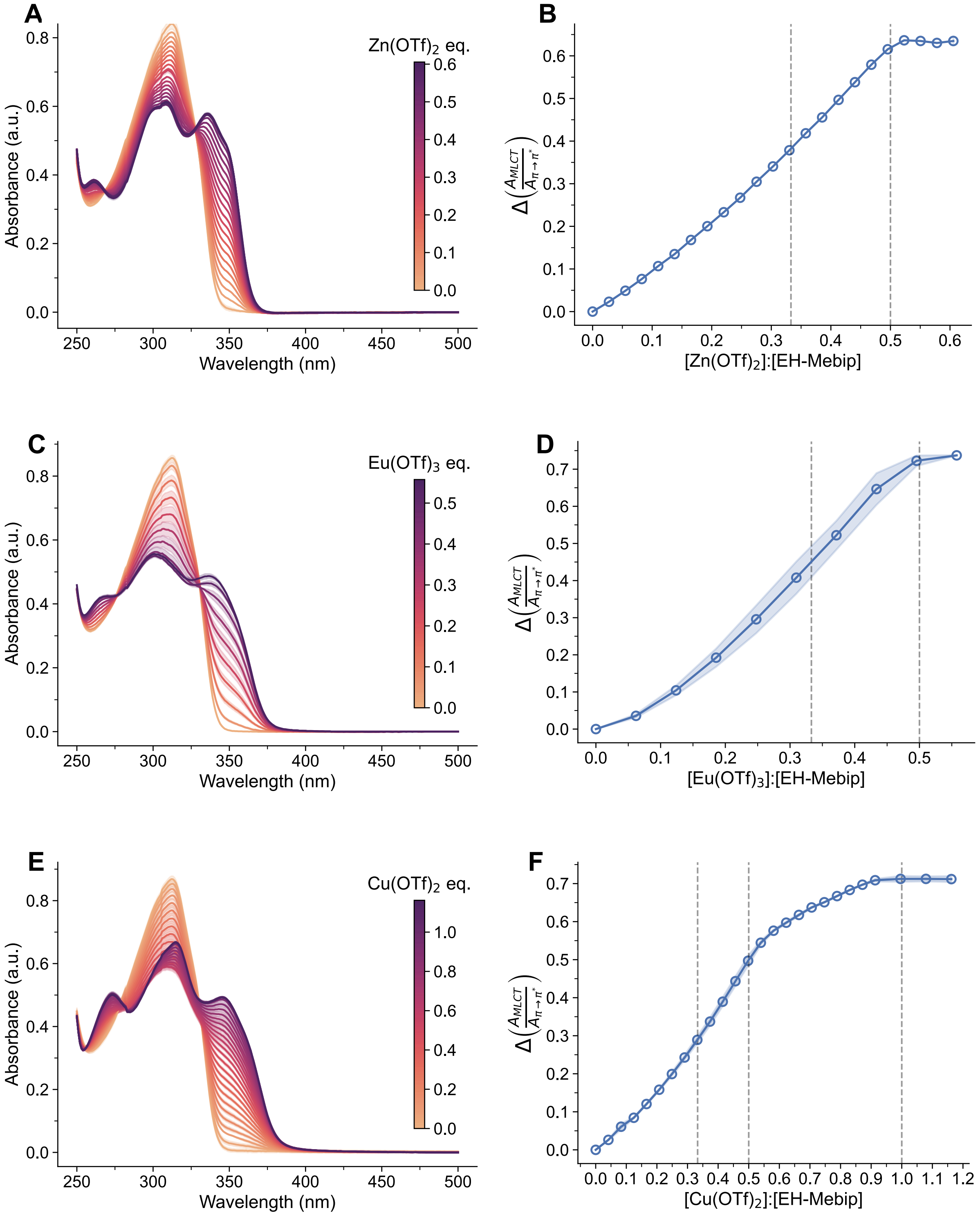

Figure S4. Titrations of solutions of the model ligand **EH-Mebip** with $Zn(OTf)_2$ (A), $Eu(OTf)_3$ (C), and $Cu(OTf)_2$ (E) in MeCN (21 μM) monitored by UV-Vis absorption spectroscopy. A non-parametric 95% confidence interval is indicated by the shading. The change in the normalized ratio of the absorbances at the maxima of the MLCT peak and the π→π* band versus the [ML complex]:[**EH-MBP**] ratio is also shown for $Zn(OTf)_2$ (B), $Eu(OTf)_3$ (D), and $Cu(OTf)_2$ (F). The scatter points are the mean of at least two repeats and a non-parametric 95 % confidence interval is indicated by the shading.

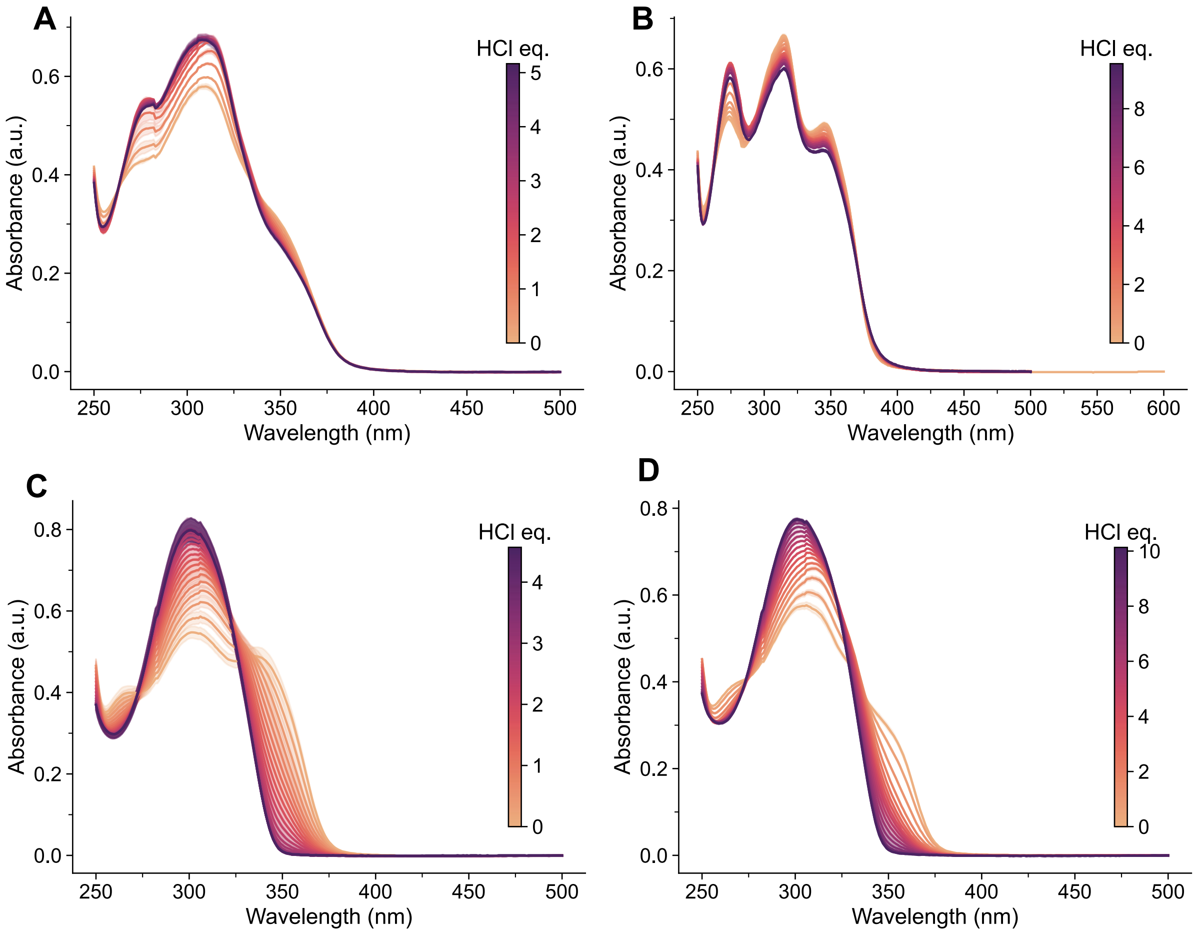

Figure S5. Titrations of solutions of the model complexes **M(EH-Mebip)$_x$** (c=11.5 µM in MeCN) with HCl (c=1.65 mM in MeCN) monitored by UV-Vis absorption spectroscopy. Shown are the absorption spectra acquired upon titration of (A) **Cu(II)(EH-Mebip)$_2$** (λ_{max} MLCT=345 nm), (B) **Cu(II)(EH-Mebip)** (λ_{max} MLCT=345 nm), (C) **Eu(EH-Mebip)$_2$** (λ_{max} MLCT=350 nm), and (D) **Eu(EH-Mebip)$_3$** (λ_{max} MLCT=350 nm).

Model studies with MBTT (S6 – S13)

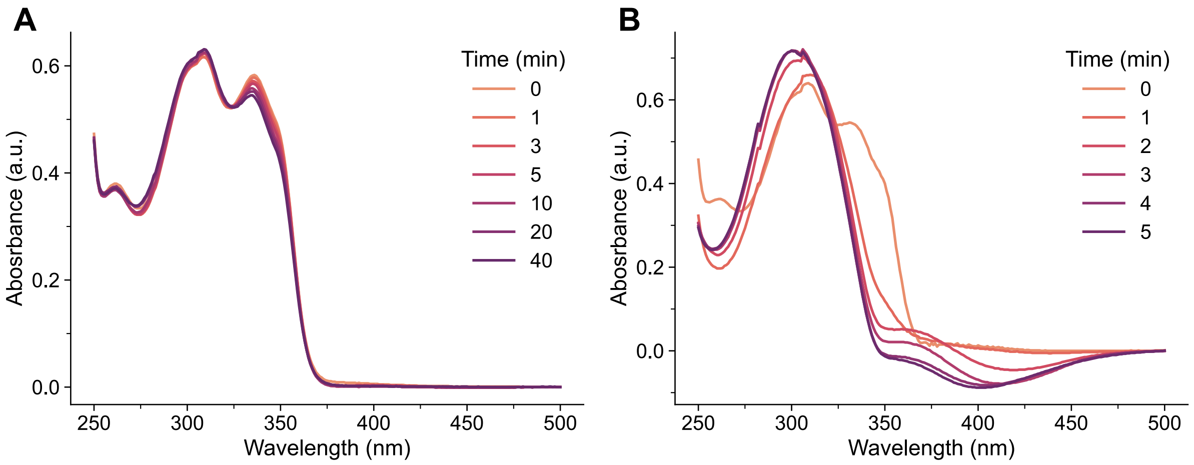

Figure S6. Decomplexation of **Zn(EH-Mebip)$_2$** through optical activation of MBTT. Solutions of **Zn(EH-Mebip)$_2$** (c=11.5 µM) and 0.17 eq (A) or 5 eq (B) MBTT in MeCN were irradiated with UV light (λ=365 nm, P=3.8 mW, power density on the sample ca. 190 mW/cm^2) and the resulting spectral changes were monitored by UV-Vis absorption spectroscopy as a function of irradiation time.

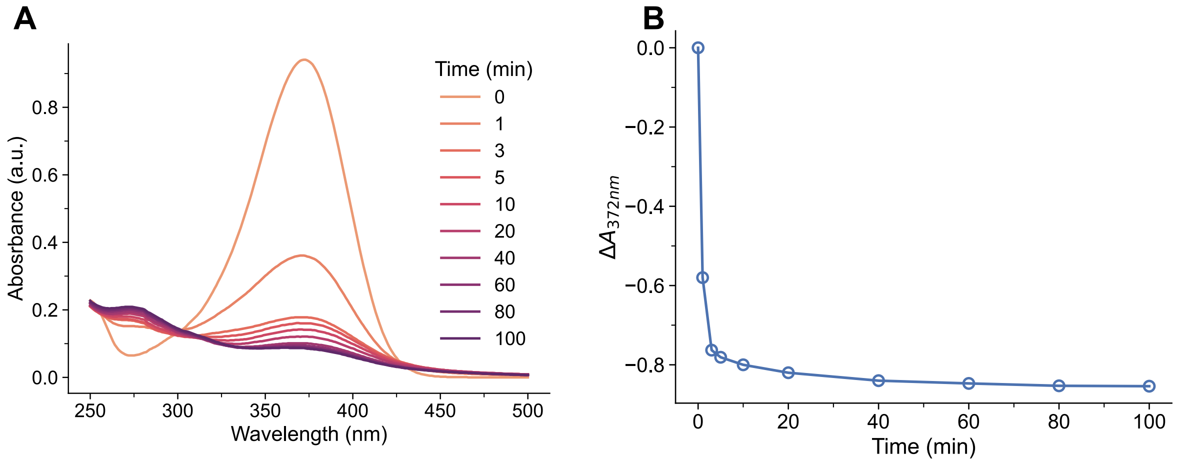

Figure S7. A solution of MBTT (c=14.5 µM) in MeCN was irradiated for periods between 1 and 100 min with UV light (λ=365 nm, P=1.29 W, power density on the sample ca. 190 mW/cm^2) and the changes were monitored offline by UV-Vis absorption spectroscopy. (A) Overlay of the spectra recorded after the different exposure times. (B) Change in absorbance at 372 nm, the peak maximum at t_0, as a function of exposure time.

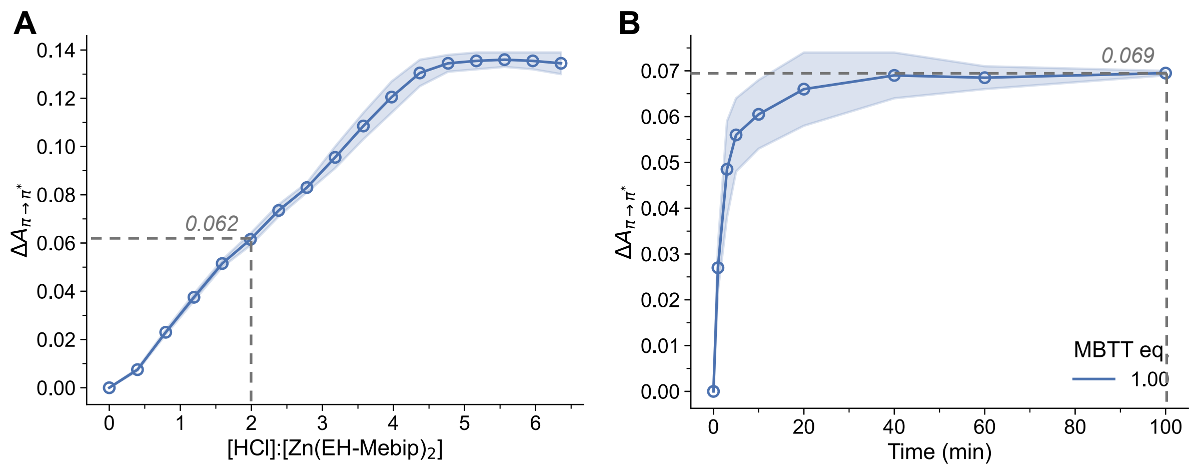

Figure S8. Comparison of the optical changes observed in the titration of **Zn(EH-Mebip)$_2$** in MeCN (c=11.5 µM) with HCl (c=1.65 mM) (A) and the decomplexation of **Zn(EH-Mebip)$_2$** through the optical activation (λ=365 nm, P=1.29 W, power density on the sample ca. 190 mW/cm^2) of MBTT (1 eq) (B) monitored by UV-Vis absorption spectroscopy. The plots show the increase in the absorbance at the maximum of the peak associated with the $\pi \rightarrow \pi^*$ band of the protonated ligand at ca. 313 nm. The absorbance change generated by optical activation of MBTT through UV exposure for 100 min is comparable to the one achieved by the addition of 2 eq of HCl.

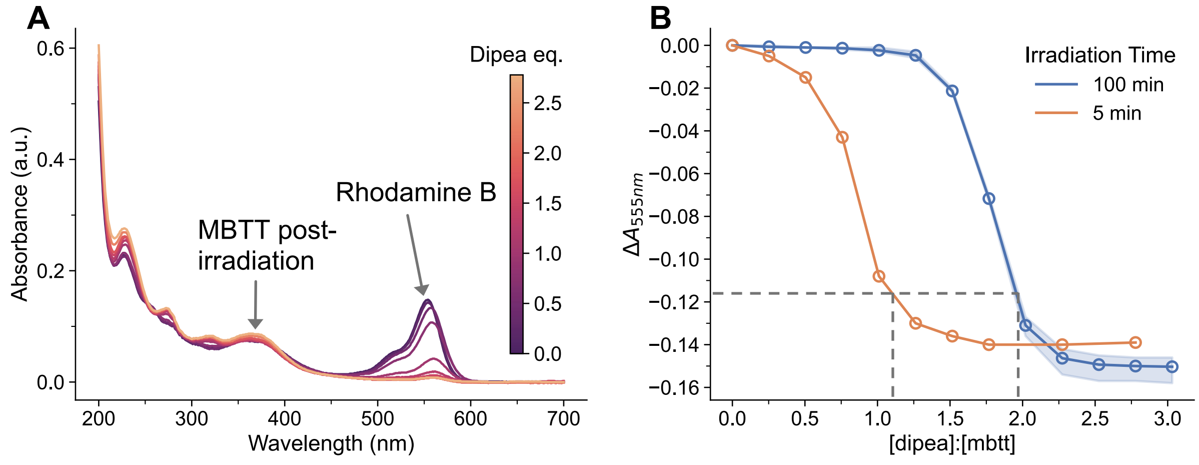

Figure S9. A solution of the basic *N,N*-diisopropylethylamine (dipea) in MeCN (c=7.18 mM), was titrated into UV-irradiated MBTT solutions in MeCN (c=9.8 µM) after the addition of Rhodamine B (c=1.31 µM). The reduction in the absorbance corresponding to opened/acidic Rhodamine B was monitored by UV-Vis spectroscopy (A) and the absorbance at 555 nm was plotted against the ratio of [dipea]:[MBTT] for different irradiation times (B). The horizontal dashed line indicates the endpoint of the titration as Rhodamine B is partially opened when introduced into MeCN.

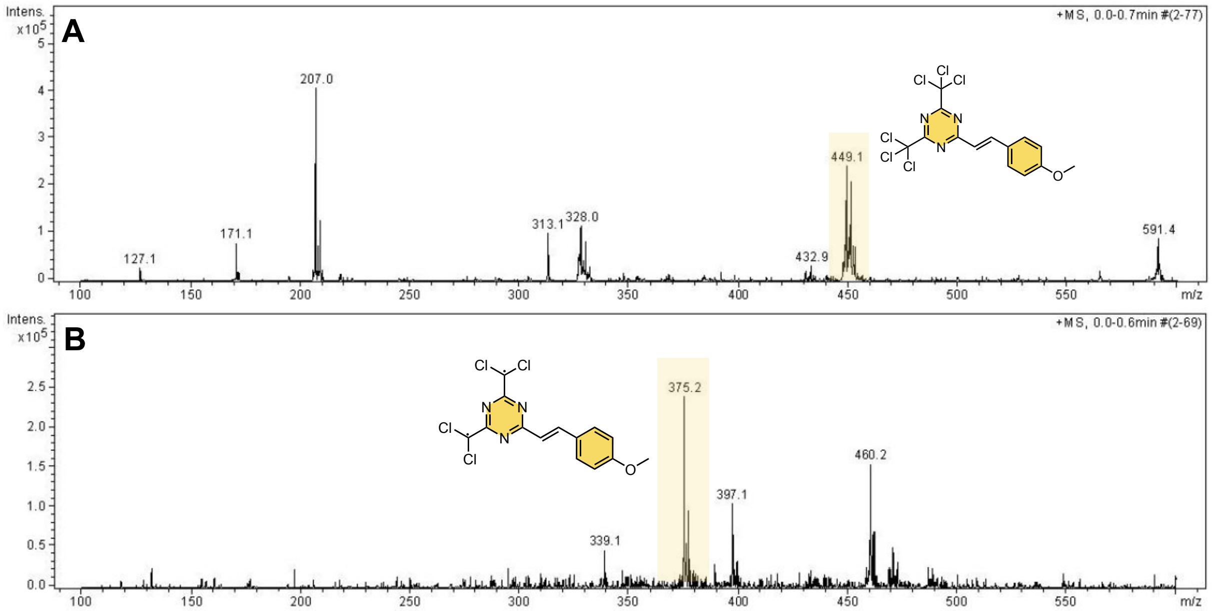

Figure S10. Mass spectra of MBTT (A) and a solution of MBTT (c=9.8 µM) in MeCN irradiated with UV (λ=365 nm, P=1.29 W, power density on the sample ca. 190 mW/cm^2) for 100 min (B). The peaks corresponding to MBTT in the unirradiated sample (A) and the fragment produced by the loss of two chlorine radicals upon irradiation (B) are highlighted.

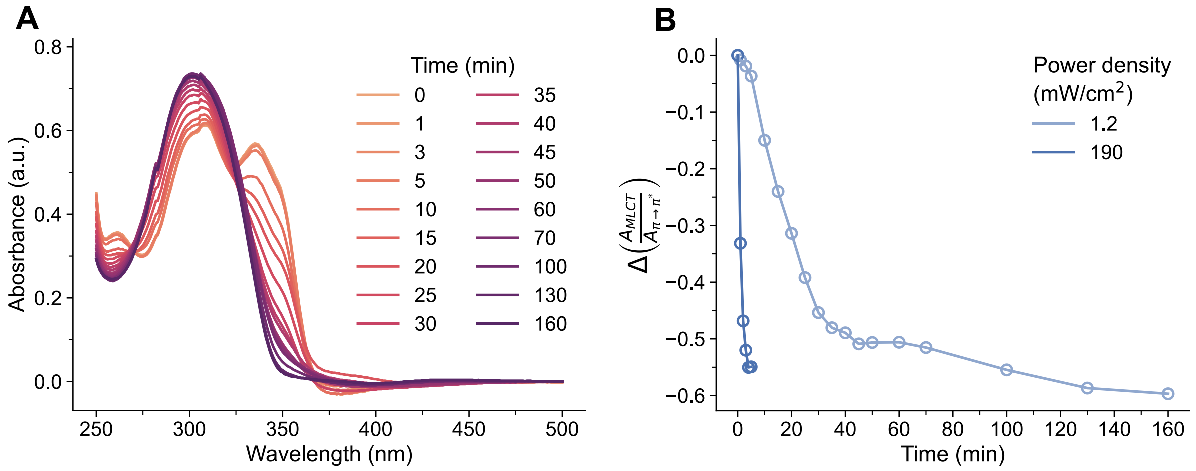

Figure S11. Decomplexation of **Zn(EH-Mebip)$_2$** through optical activation of MBTT. A solution of **Zn(EH-Mebip)$_2$** (c=11.5 µM) and MBTT (5 eq) in MeCN was irradiated with diffuse UV light (λ=365 nm, P=6 W, power density on the sample ca. 1.2 mW/cm^2) or more intense UV light (λ=365 nm, P=1.29 W, power density on the sample ca. 190 mW/cm^2) and the resulting spectral changes were monitored by UV-Vis absorption spectroscopy. (A) Plot showing the evolution of the absorption spectra with irradiation time with diffuse UV light. (B) Plot of the ratio of the absorbances at 335 nm (MLCT band of **Zn(EH-Mebip)$_2$**) and peak maxima around 313 nm ($\pi\rightarrow\pi^*$ band of the protonated ligand) as a function of irradiation time for two different power densities (data taken from Figure S10a and Figure 4a).

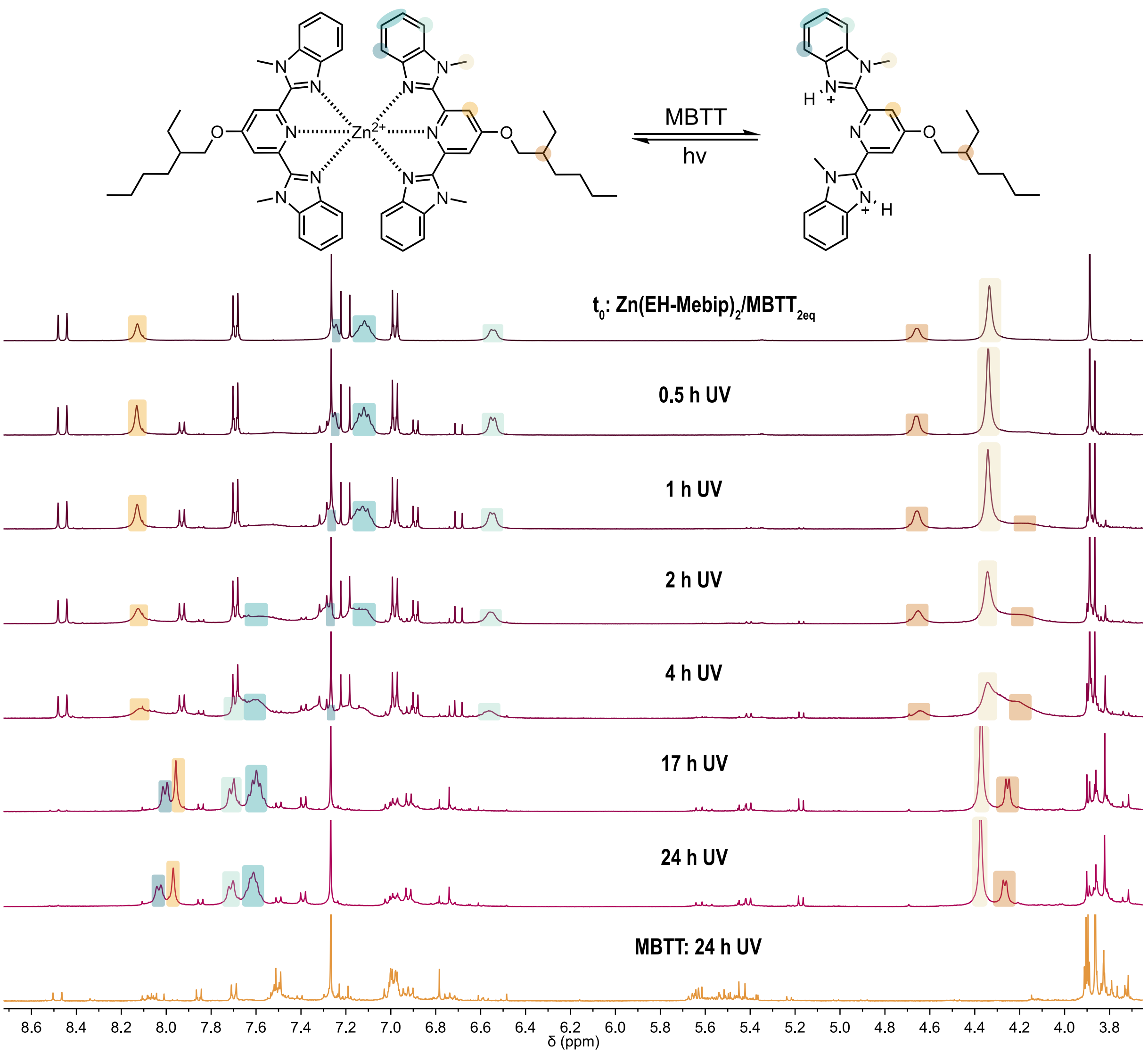

Figure S12. ^{1}H-NMR spectra demonstrating the decomplexation of **Zn(EH-Mebip)$_2$** upon optical activation of MBTT. A solution of **Zn(EH-Mebip)$_2$** (c=17.5 mM) and MBTT (2 eq, 1.75 mM) in $CDCl_3$ was irradiated with diffuse UV light (λ=365 nm, P=6 W, power density on the sample ca. 1.2 mW/cm^2) and ^{1}H-NMR spectra were recorded after irradiation for the times indicated. Relevant peaks of the complex and the protonated free ligand are highlighted and assigned per color code in the chemical structures shown. The spectrum of a control experiment in which a solution of MBTT in $CDCl_3$ was irradiated for 24 h with UV light under the same conditions is also shown. To enable these experiments, the concentrations of **Zn(EH-Mebip)$_2$** and MBTT were increased by ca. 3 orders of magnitude compared to UV/Vis experiments, $CDCl_3$ was used as the solvent, and (on account of the limited solubility of MBTT) the ratio of [MBTT]:[**Zn(EH-Mebip)$_2$**] was set to 2. The spectra show a gradual decrease in the intensity of resonances related to **Zn(EH-Mebip)$_2$** and a concomitant increase in the intensity of signals corresponding to the free protonated ligand upon irradiation. At the same time, signals in the aromatic region pertaining to MBTT shift and multiply because of PAG decomposition.

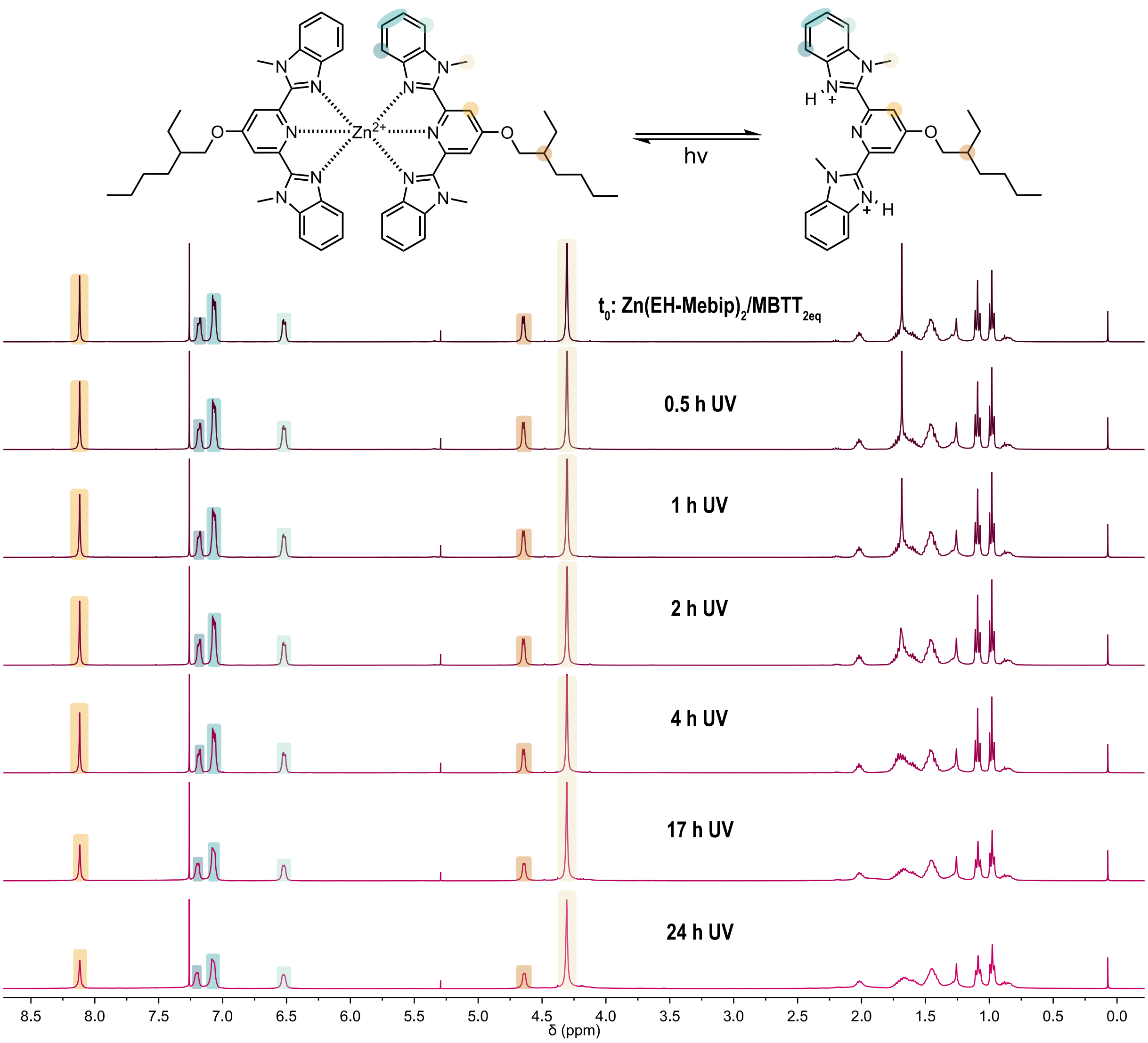

Figure S13. A solution of the complex **Zn(EH-Mebip)$_2$** (17.2 mM) in $CDCl_3$ was irradiated with diffuse UV light (λ=365 nm, P=6 W, power density on the sample ca. 1.2 mW/cm^2) and ^{1}H-NMR spectra were recorded after irradiation for the times indicated. Peaks pertaining to the complex are highlighted.

MSP investigations (Scheme S1, Table S1, S14 – S22)

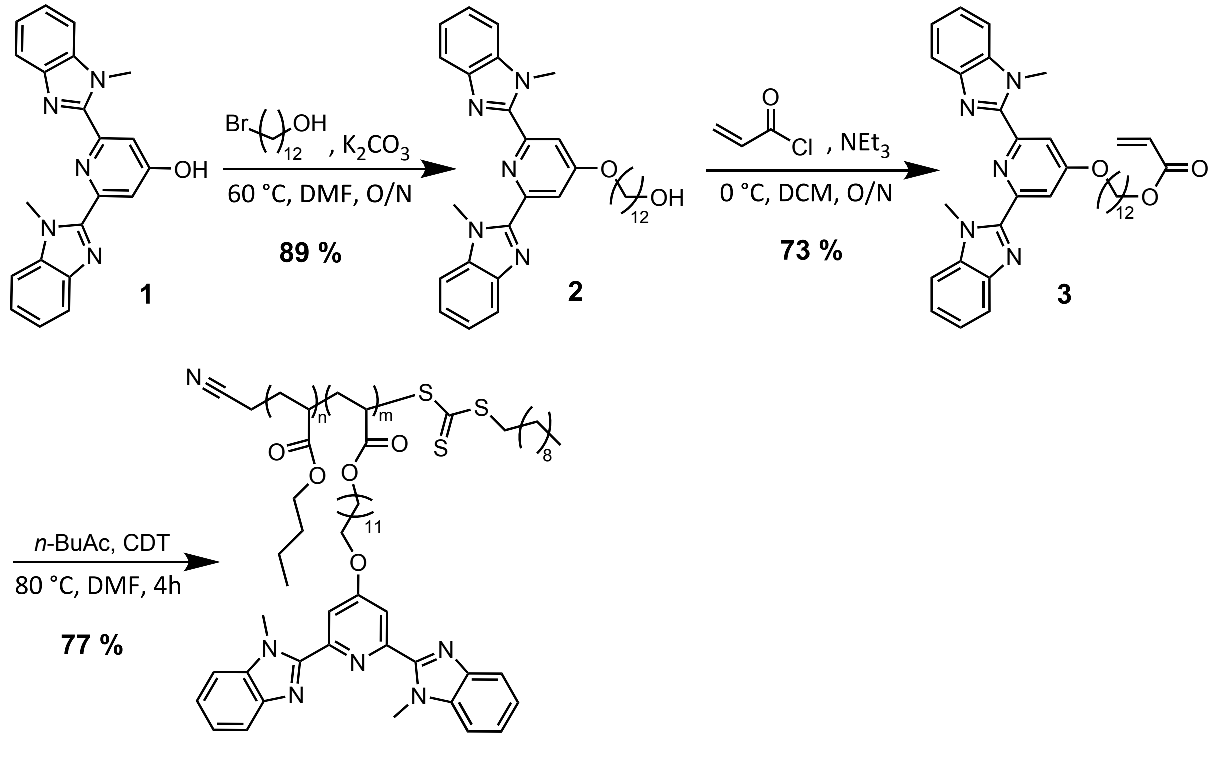

PBA-*co*-MBA$_{xx}$-YY

Scheme S1. Synthesis of Mebip-acrylate **3** and RAFT *co*-polymerization of **3** and *n*-butyl acrylate to afford *co*-polymers of **PBA-*co*-MBA$_{xx}$-YY**.

Table S1. Pertinent properties of the poly(*n*-butyl acrylate-*co*-Mebip acrylate)s prepared.

Name	M_n (g/mol) [a]	Mebip in polymer (mol%) [b]	Mebip in feed (mol%) [c]	Đ [a]	Mebip/Chain	Swelling[d]
**PBA-*co*-MBA$_6$-13**	13’030	5.8	4.8	1.1	5	Soluble
**PBA-*co*-MBA$_5$-43**	42’509	4.5	4.8	1.3	14	Robust gel
**PBA-*co*-MBA$_{10}$-29**	28’530	10.0	9.9	1.7	16	Poor swelling

[a]Measured by Size Exclusion Chromatography (SEC).
[b]Measured by ^{1}H-NMR spectroscopy.
[c]Calculated from feed composition.
[d]In $CHCl_3$. After adding stoichiometric amounts of $Zn(OTf)_2$ to the polymer.

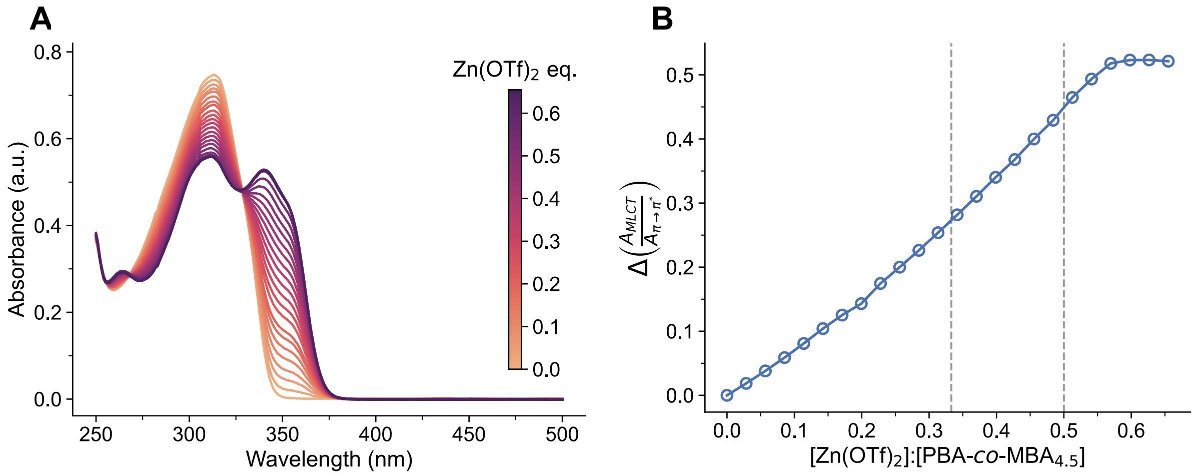

Figure S14. Titration of a solution of **PBA-*co*-MBA$_5$-43** (*c*=20 μM of Mebip in MeCN) with aliquots of Zn(OTf)$_2$ monitored by UV-Vis absorption spectroscopy. (A) Overlay of the spectra acquired as a function of [Zn(OTf)$_2$]:[ligand residues]. (B) Plot of the change in the ratio of the absorbances at the maxima of the MLCT band and the π→π* band versus the [Zn(OTf)$_2$]:[ligand residue] ratio (data extracted from (A)).

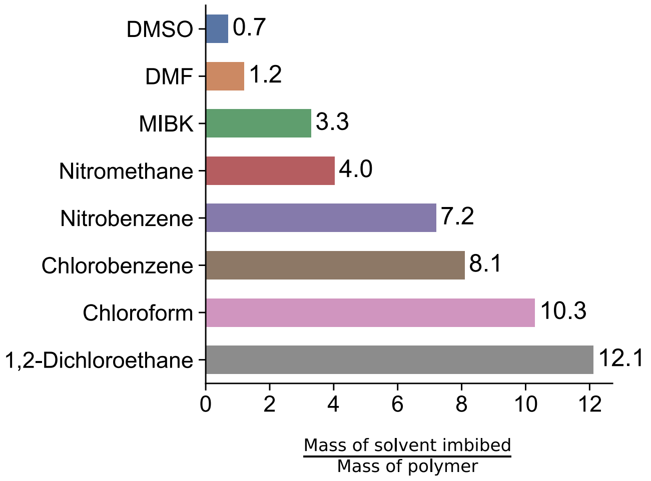

Figure S15. A solvent screening was performed by swelling the dried MSP network made from **Zn(PBA-co-MBA$_5$-43)** with the solvents listed. The mass fraction of solvent taken up after immersing the sample in the respective solvent and equilibrium swelling is recorded for each solvent investigated.

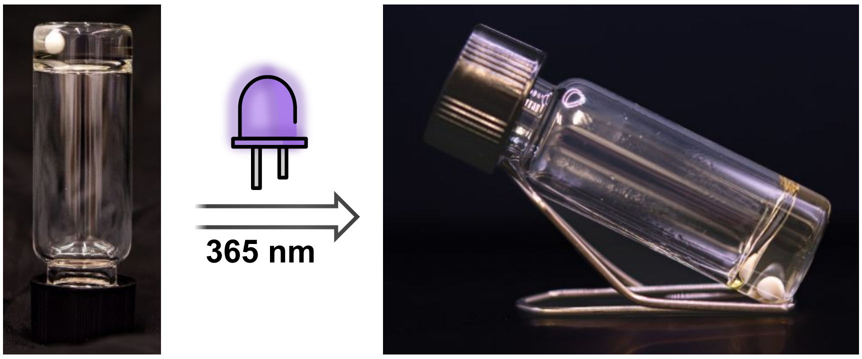

Figure S16. Pictures of a **Zn(PBA-*co*-MBA$_5$-43)** gel in chlorobenzene before (left) and after (right) irradiation at 365 nm for 10 min with a power density on the sample of 90 mW/cm^2. The gel contains 12 wt% of the MSP (relative to the solvent).

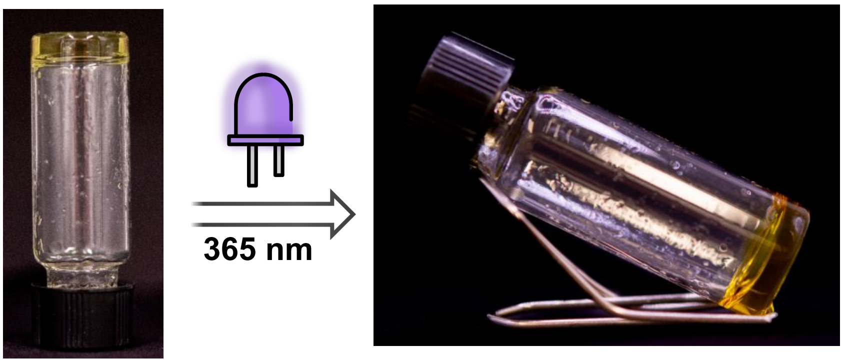

Figure S17. Pictures of a **Zn(PBA-*co*-MBA$_5$-43)/MBTT** gel in chlorobenzene before (left) and after (right) irradiation at 365 nm for 20 min at 90 mW/cm^2. The gel contains 12 wt% of the MSP (relative to the solvent) and 0.12 wt% of MBTT (relative to the solvent, corresponding to an [MBTT]:[**Zn(MBA)$_2$**] ratio of 0.15).

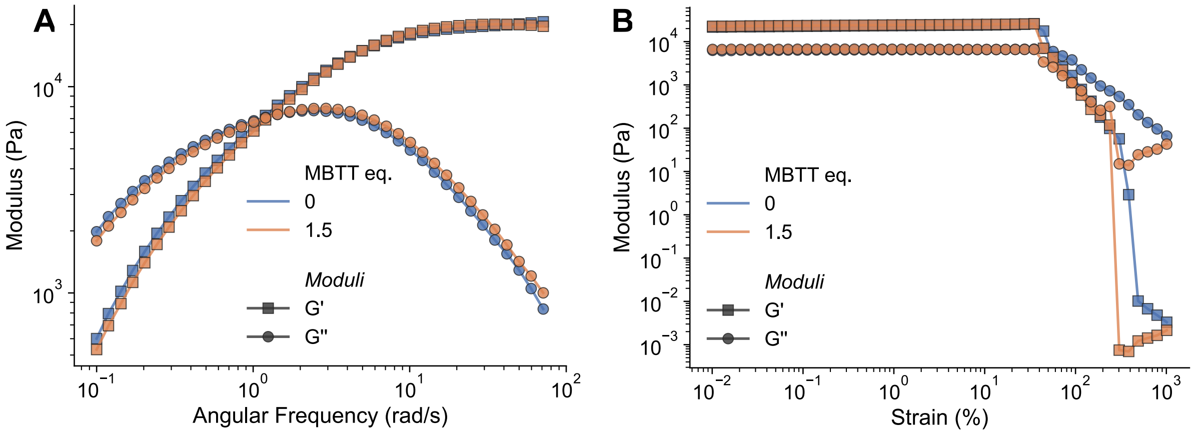

Figure S18. Oscillatory shear rheology of **Zn(PBA-*co*-MBA$_5$-43)** and **Zn(PBA-*co*-MBA$_5$-43)/MBTT** gels in chlorobenzene. (A) Frequency sweeps carried out at γ = 1%. (B) Amplitude sweeps carried out at ω = 10 rad/s. Both gels contain 12 wt% of the MSP and the MBTT gel additionally 1.2 wt% of MBTT ([MBTT]:[**Zn(MBA)$_2$**] = 1.5). The experiments were conducted under isothermal conditions at 25 °C.

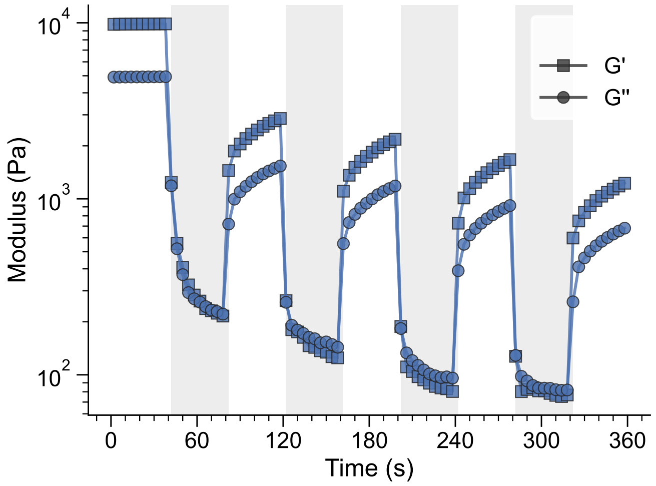

Figure S19. Oscillatory shear rheology of a **Zn(PBA-*co*-MBA$_5$-43)** gel in chlorobenzene (12 wt% of the MSP). The time sweep was carried out with a constant frequency of ω = 10 rad/s and the applied strain was switched between 1 and 150%, *i.e.*, values well below and above γ_{crit}.

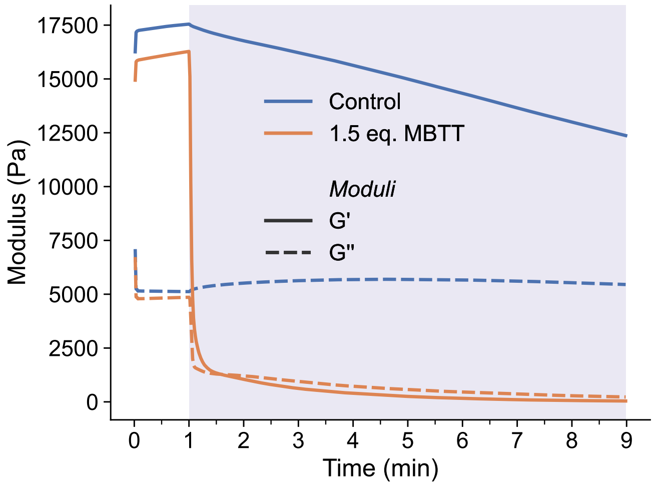

Figure S20. Optorheological experiments (time sweeps) of **Zn(PBA-*co*-MBA$_5$-43)** and **Zn(PBA-*co*-MBA$_5$-43)/MBTT** gels in chlorobenzene. After an idle period of 1 min, the UV light was switched on (purple shade). Both gels contain 12 wt% of the MSP and the MBTT gel additionally 1.2 wt% of MBTT ([MBTT]:[**Zn(MBA)$_2$**] = 1.5). The experiments were conducted under nominally isothermal conditions at 25 °C with γ = 1%, ω = 10 rad/s, λ=385 nm, and a power density on the samples of ca. 190 mW/cm^2. The data are identical to the ones shown in Figure 6a in the main manuscript but are plotted with a linear y-axis.

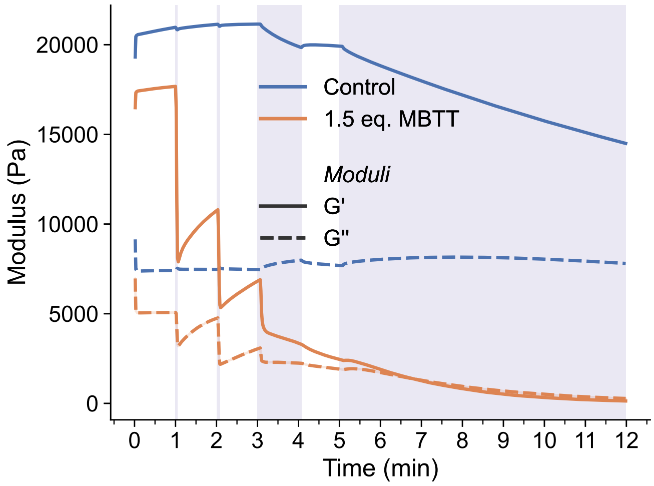

Figure S21. Optorheological experiments (time sweeps) of **Zn(PBA-*co*-MBA$_5$-43)** and **Zn(PBA-*co*-MBA$_5$-43)/MBTT** gels in chlorobenzene. After an idle period of 1 min, the UV light was switched on for periods of 1 s, 1 s, 1 min, and 7 min (purple shades). Both gels contain 12 wt% of the MSP and the MBTT gel additionally 1.2 wt% of MBTT ([MBTT]:[**Zn(MBA)$_2$**] = 1.5). The experiments were conducted under nominally isothermal conditions at 25 °C with γ = 1%, ω = 10 rad/s, λ=385 nm, and a power density on the samples of ca. 190 mW/cm^2. The data are identical to the ones shown in Figure 6b in the main manuscript but are plotted with a linear y-axis.

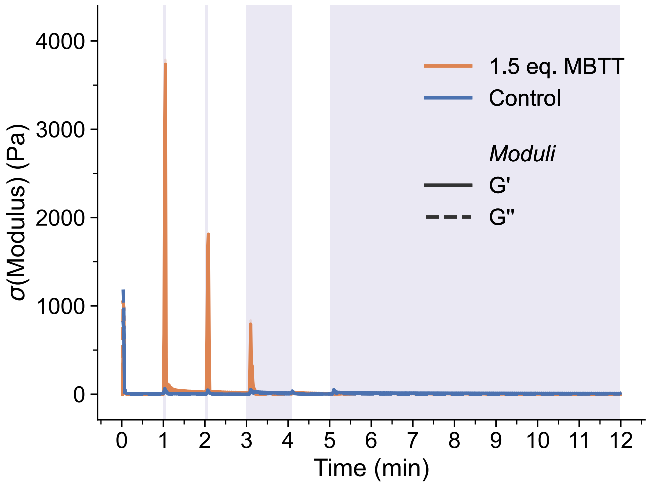

Figure S22. A rolling standard deviation, with a 2-second window, of the storage (G') and loss (G'') modulus was developed from the data shown in Figures 6b and S20. Sharp changes in standard deviation indicate an immediate deviation from the mean signal, reflecting an instant response to UV-light exposure for the **Zn(PBA-*co*-MBA$_5$-43)/MBTT** gel. No deviation is observed for the MBTT-free **Zn(PBA-*co*-MBA$_5$-43)** gels.

Materials and Instrumentation

Materials. Solvents were purchased as reagent grade and used as received (Sigma-Aldrich or Fisher Scientific). Zinc triflate ($Zn(OTf)_2$) and copper(II) triflate ($Cu(OTf)_2$) were used as received from STREM Chemicals. Europium(III) triflate ($Eu(OTf_2)_2$) (Sigma-Aldrich), cyanomethyl dodecyl trithiocarbonate (Sigma-Aldrich), chelidamic acid (Intatrade Chemicals GmbH), acryloyl chloride (Sigma-Aldrich), 5-hydroxypyridine-2-carboxylic acid (Fluorochem) and other reagents were used as received. 2,2'-Azobis(2-methylpropionitrile) (AIBN, Sigma-Aldrich) was recrystallized from hot ethanol prior to use. 2-(4-Methoxystyryl)-4,6-bis(trichloromethyl)-1,3,5-triazine (MBTT) was dissolved in acetone and passed over basic Al_2O_3 prior to use to remove residual acid; acetone was removed in *vacuo*. The inhibitor was removed from *n*-butyl acrylate by passing over basic Al_2O_3 prior to use. Column chromatography was performed on a Biotage Isolera One instrument with Biotage SNAP columns where UV-light of 254 nm wavelength was used for detection. Three different UV light sources were used in experiments and indicated accordingly; A Thorlabs-purchased UV-mounted LED (M365L3, λ=365 nm, P=1.29 W, power density on the sample ca. 190 mW/cm^2) with a collimated lens was controlled with an LEDD1B driver and used for samples held in cuvettes, Tattu UV Torches (λ=365 nm, P=5 W, power density on the sample ca. 90 mW/cm^2) were used for the irradiation of vialed samples, and a TLC lamp (λ=365 nm, P=6 W, power density on the sample ca. 1.2 mW/cm^2) was used as a diffuse, low-intensity UV light source. A Hönle UV-meter with a flat UVA sensor was used to measure all power densities recorded.

Ultraviolet Visible (UV/Vis) Absorption Spectroscopy. A Shimadzu UV-2401 PC spectrophotometer was used to conduct UV/Vis measurements of solutions in quartz cuvettes of 1 cm path length. Measurements were conducted at room temperature. Python code, using open-source packages, was written to parse, analyze, and plot data.

Nuclear magnetic resonance (NMR) spectroscopy. NMR spectra were recorded on a Bruker Avance DPX 400 spectrometer (400 MHZ for ^{1}H, 100 MHz for ^{13}C). Deuterated solvents were used in all cases, and spectra were calibrated to the residual solvent peak or tetramethylsilane (TMS) if the solvent peak was not identified. MestReNova (12.0.2) software was used to analyze the data. The multiplicities are abbreviated accordingly as s: singlet, d: doublet, dd: doublet of doublet, t: triplet, q: quintet, m: multiplet, br: broad signal.

Size Exclusion Chromatography (SEC). SEC was conducted with an Agilent Technologies instrument (1200 HPLC) in THF (flow rate 1.0 mL/min, 40 °C), a Polymer Laboratories 5 µm mixed-C guard column, and two SEC columns. A refractive index and a UV detector (Optilab REX interferometric refractometer, miniDawn TREOS laserphotometer) were used. Calibration was performed with poly(methyl methacrylate) (PMMA) standards.

Fourier-Transform Infrared (FTIR) spectroscopy. A Perkin Elmer Spectrum 65 spectrometer was used for the measurement of dry samples (4000-60 cm^{-1}, 4 cm^{-1}, 8 scans/sample). Python code, using open-source packages, was written to parse, analyze, and plot data.

Rheology. An Anton Paar MCR 702 MultiDrive rheometer was used to perform measurements. Samples were analyzed at room temperature (ca. 25°C) using a roughened 8 mm upper parallel plate within a sealed chamber and incubated for 1 min prior to measuring. Measurements were repeated to ensure reproducibility with different batches of gel. For strain sweeps, a 10 rad/s angular frequency was applied while varying the strain from 0.1 to 1000% at a gap height of 1.5 mm. Frequency sweeps were conducted at 1% strain while varying the angular frequency from 600 rad/s to 0.1 rad/s at a gap height of 1.5 mm. Self-healing characterization was performed at 10 rad/s while alternating the strain between 1 and 150% for 5 cycles at a gap height of 1.5 mm. *In-situ* UV irradiation experiments were performed with an Omnicure LX500

controller powering a 385 nm LED head through a quartz bottom plate. Samples were irradiated at a measured power density of 146 mW/cm^2 at a gap height of 0.5 mm. Python code, using open-source packages, was written to parse, analyze, and plot data.

Synthetic Methods and Characterization

6-(1-Methyl-1H-benzo[d]imidazol-2-yl)pyridin-3-ol (MBP)

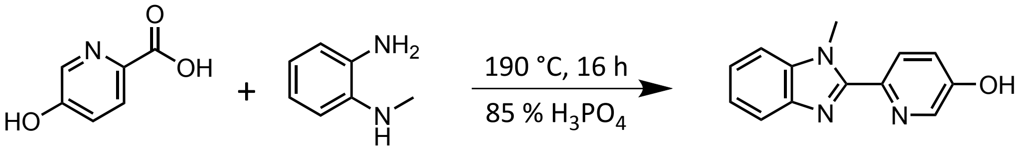

To a 100 mL round bottom flask, 5-hydroxypyridine-2-carboxylic acid (10.00 g, 71.89 mmol) was added and dissolved in 50 mL of 85% H_3PO_4. While stirring, *N*-methyl-1,2-phenylenediamine (17.53 g, 89.86 mmol, 1.25 eq.) was added and the solution was subsequently degassed by sparging with N_2 for 30 min. The reaction mixture was heated to 200 °C and stirred under N_2 under reflux overnight. The reaction mixture was then cooled to RT, dissolved in H_2O (40 mL) and sodium citrate was added for buffering purposes. To the solution concentrated NH_3 (ammonium hydroxide) was added in a dropwise fashion until pH 6 was reached. The solution was then placed in the fridge overnight, the precipitate was filtered off, washed with H_2O, and dried in *vacuo* at 60 °C to afford a gray solid (10.34 g, 63 %).

^{1}H NMR (300 MHz, DMSO-d$_6$): δ 11.37 (s, 1H), 7.79 (s, 2H), 7.78 – 7.73 (m, 2H), 7.70 – 7.65 (m, 2H), 7.39 – 7.26 (m, 4H), 4.25 (s, 6H). HRMS (ESI): calcd. for $[M]^+$ 225.09 and $[M + Na]^+$ 248.08, found: 226 and 247.9.^{13}C NMR (101 MHz, DMSO-d$_6$): δ 154.4, 150.2, 142.1, 141.1, 137.0, 136.8, 125.6, 123.2, 122.6, 122.5, 122.0, 118.9, 110.4, 32.5. HRMS (ESI): calcd. for $[M]^+$ 225.09 and $[M + Na]^+$ 248.08, found: 226.0 and 247.9.

2-(5-((2-Ethylhexyl)oxy)pyridin-2-yl)-1-methyl-1H-benzo[d]imidazole (EH-MBP)

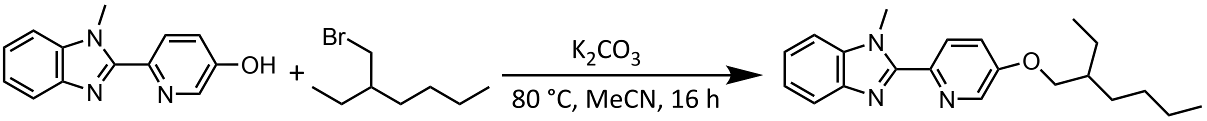

A round bottom flask was charged with **MBP** (1.00 g, 4.44 mmol) and 35 mL of MeCN. To the stirred solution, an excess of K_2CO_3 was added in portions, before 2-ethylhexyl bromide (1.18 mL, 6.66 mmol, 1.5 eq.) was added dropwise. The reaction mixture was then stirred under reflux overnight, cooled to RT, filtered, and the solvent was removed in *vacuo*. The product was purified by column chromatography (DCM/MeOH 100:0 to 97:3) to afford an off-white solid that was dried at 60 °C in *vacuo* (1.03 g, 68%).

^{1}H NMR (400 MHz, $CDCl_3$): δ 8.30 (dd, *J* = 2.9, 0.7 Hz, 2H), 8.25 (dd, *J* = 8.8, 0.6 Hz, 2H), 7.77 – 7.69 (m, 2H), 7.38 – 7.30 (m, 2H), 7.31 – 7.19 (m, 7H), 4.17 (s, 6H), 3.90 (dd, *J* = 5.7, 1.0 Hz, 4H), 1.71 (p, *J* = 6.1 Hz, 3H), 1.55 – 1.32 (m, 9H), 1.33 – 1.16 (m, 11H), 0.94 – 0.79 (m, 13H).^{13}C NMR (101 MHz, $CDCl_3$): δ 155.9, 150.7, 143.0, 142.7, 137.4, 136.9, 125.7, 123.0, 122.5, 121.7, 119.8, 109.8, 71.2, 39.5, 32.8, 30.6, 29.2, 23.9, 23.2, 14.2, 11.3. HRMS (ESI): calcd. for $[M]^+$ 337 and $[M + Na]^+$ 359.9, found: 338 and 360.

2,6-bis(1′-Methylbenzimidazolyl)-4-hydroxypyridine (1)

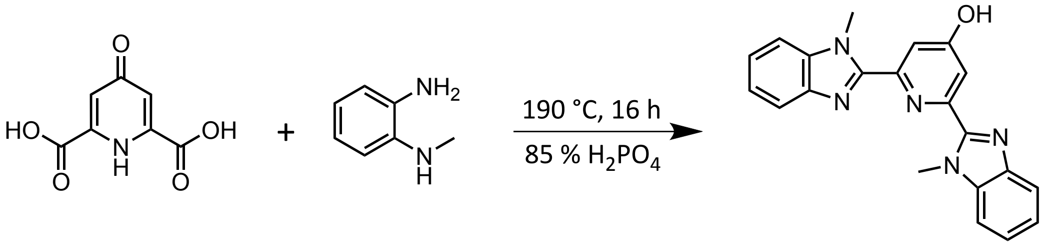

2,6-Bis(1′-methylbenzimidazolyl)-4-hydroxypyridine **(1)** was prepared according to a method reported in the literature.[1]

^{1}H NMR (300 MHz, DMSO-d$_6$): δ 11.37 (s, 1H), 7.79 (s, 2H), 7.78 – 7.73 (m, 2H), 7.70 – 7.65 (m, 2H), 7.39 – 7.26 (m, 4H), 4.25 (s, 6H). ^{13}C NMR (101 MHz, DMSO-d$_6$): δ 165.1, 150.9, 149.9, 142.0, 137.0, 123.1, 122.3, 119.4, 112.4, 110.8, 32.5. HRMS (ESI): calcd. for $[M]^+$ 356.15 and $[M + Na]^+$ 378.13, found: 356.0 and 378.0.

Bis(1'-methylbenzimidazoyl)-4-(2-ethylhexyloxy)pyridine (EH-Mebip)

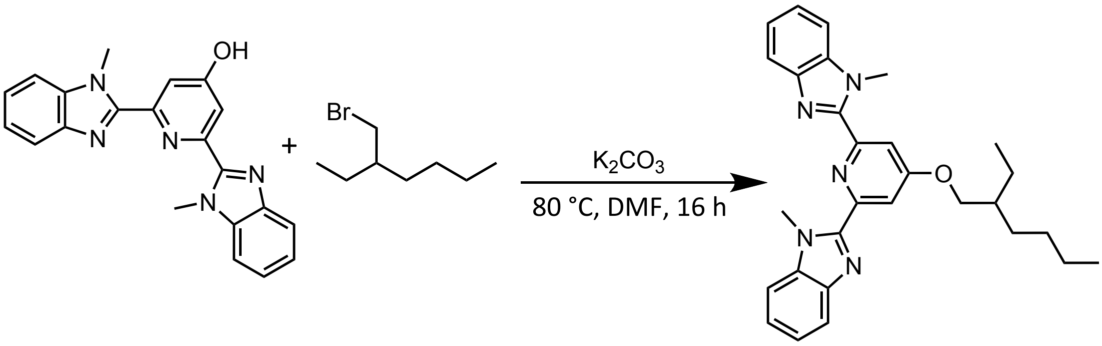

Bis(1'-methylbenzimidazoyl)-4-(2-ethylhexyloxy)pyridine was prepared by adapting a reported method.[2] A round bottom flask was charged with **1** (1.00 g, 2.81 mmol) and 25 mL of dry DMF. K_2CO_3 was added in portions until a pinkish suspension was obtained. To the stirred mixture, 2-ethylhexyl bromide (1.18 mL, 6.66 mmol, 1.5 eq.) was added dropwise. The reaction mixture was then stirred under reflux overnight under argon, cooled to RT, poured into 100 mL of H_2O, and the mixture was extracted with EtOAc (3 x 100 mL). The organic layer was washed with saturated NH_4Cl (100 mL) and dried over $MgSO_4$. After filtration, the solvent was removed in *vacuo* and the resulting solid was recrystallized in DCM/Hexane (70/30 v/v); after cooling in a refrigerator for several hours, the crystals were filtered off, and washed with hexane, and dried in *vacuo* at 60 °C to obtain an off-white solid (0.95 g, 70%).

^{1}H NMR (400 MHz, $CDCl_3$): δ 7.87 (s, 2H), 7.84 – 7.74 (m, 2H), 7.42 – 7.35 (m, 2H), 7.38 – 7.23 (m, 4H), 4.17 (s, 6H), 4.05 (dd, J = 5.5, 1.4 Hz, 2H), 1.72 (hept, J = 6.2 Hz, 1H), 1.52 – 1.30 (m, 4H), 1.26 (ddd, J = 8.6, 4.6, 2.8 Hz, 4H), 0.92 – 0.76 (m, 6H).^{13}C NMR (101 MHz, $CDCl_3$): δ 167.1, 150.4, 137.1, 123.9, 123.2, 120.1, 112.3, 110.1, 71.2, 39.4, 32.7, 30.6, 29.2, 24.0, 23.2, 14.2, 11.3. HRMS (ESI): calcd. for $[M]^+$ 467.21 and $[M + Na]^+$ 490.2, found: 468.1 and 490.0.

12-((2,6-Bis(1-methyl-1*H*-benzo[*d*]imidazol-2-yl)pyridin-4-yl)oxy)dodecan-1-ol 2 (2)

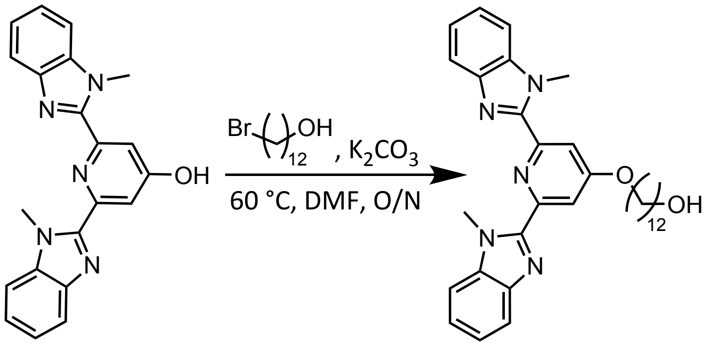

A round bottom Schlenk flask was charged with **1** (7.00 g, 19.70 mmol) and anhydrous DMF (200 mL). The solution was stirred at 60 °C under N_2 and K_2CO_3 was added until a light pinkish suspension had formed. To the stirred suspension, 12-bromo-1-dodecanol (8.4 g, 31.67 mmol) dissolved in a minimal amount of anhydrous DMF was added dropwise. The reaction mixture was stirred overnight at 60 °C under argon. The solvent was removed in vacuo, the resulting residue was redissolved in DCM, and the solution was filtered. The solvent was removed in *vacuo* and the product was crystallized in DCM/hexane (70/30 v/v) to obtain pink crystals. The filtrate was concentrated and the remaining solid was recrystallised again from DCM/hexane. The combined crystal fractions were dried in vacuo at 60 °C and the product was obtained as a pink/off white solid (8.19 g, 77%).

^{1}H NMR (400 MHz, $CDCl_3$-d): δ 7.85 (s, 2H), 7.81 – 7.76 (m, 2H), 7.41 – 7.35 (m, 2H), 7.33 – 7.24 (m, 4H), 4.16 (s, 8H), 3.57 (q, J = 6.2 Hz, 2H), 1.79 (q, J = 7.0 Hz, 2H), 1.58 – 1.36 (m, 8H), 1.33 – 1.16 (m, 15H). ^{13}C NMR (101 MHz, $CDCl_3$): δ 166.8, 151.2, 150.6, 142.6, 137.3, 123.7, 123.0, 120.3, 112.0, 110.0, 68.8, 63.1, 33.0, 32.7, 29.6, 29.6, 29.5, 29.5, 29.3, 29.01, 26.0, 25.9. HRMS (ESI): calcd. for $[M + Na]^+$ 562.23 and found 562.1.

12-((2,6-Bis(1-methyl-1*H*-benzo[*d*]imidazol-2-yl)pyridin-4-yl)oxy)dodecyl acrylate (3, MBA)

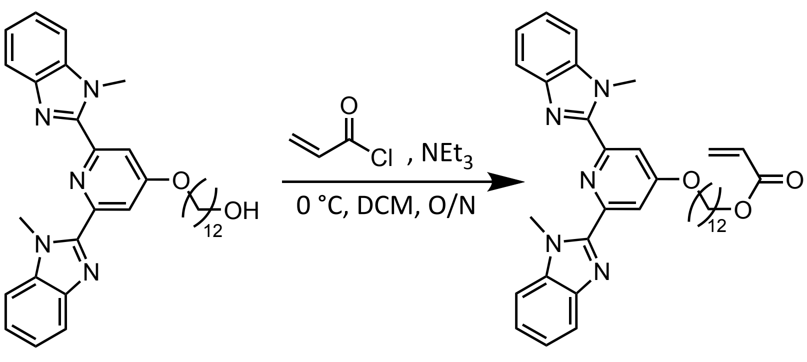

To a round bottom flask, **2** (5.00 g, 9.26 mmol) was added and anhydrous DCM was added under N_2 flow. Triethylamine (5.17 mL, 37.06 mmol) was added, and the reaction mixture was stirred at room temperature for 15 min. The reaction flask was then placed in an ice bath, and after the reaction mixture had cooled, acryloyl chloride was added (1.66 mL, 20.0 mmol). The mixture was then stirred for 16 h and allowed to reach room temperature under an N_2 or argon atmosphere; the solvent was removed, the solid residue obtained was suspended in THF, and the mixture was filtered. The filtrate was redissolved in DCM, the solution was washed 2 x with $NaHCO_3$ and 1 x with brine, and finally dried over Na_2SO_4. After evaporating the solvent in *vacuo*, the product was purified via column chromatography (silica gel, DCM/MeOH 99:1–97:3 v/v). An off-white solid was obtained (4.40 g, 80%).

^{1}H NMR (400 MHz, $CDCl_3$): δ 7.93 (s, 2H), 7.89 – 7.84 (m, 2H), 7.48 – 7.43 (m, 2H), 7.40 – 7.31 (m, 4H), 6.39 (dd, *J* = 17.3, 1.5 Hz, 1H), 6.11 (dd, *J* = 17.3, 10.4 Hz, 1H), 5.80 (dd, *J* = 10.4, 1.6 Hz, 1H), 4.23 (s, 8H), 4.14 (t, *J* = 6.7 Hz, 2H), 1.85 (p, *J* = 6.6 Hz, 2H), 1.66 (dq, *J* = 8.0, 6.6 Hz, 2H), 1.55 – 1.44 (m, 2H), 1.42 – 1.24 (m, 15H). ^{13}C NMR (101 MHz, $CDCl_3$): δ 166.8, 166.5, 151.2, 150.6, 142.7, 137.3, 130.5, 128.8, 123.7, 122.9, 120.3, 112.0, 110.0, 68.83, 64.9, 32.7, 29.7, 29.6, 29.4, 29.0, 28.8, 26.1, 26.0. HRMS (ESI): calcd. for $[M + Na]^+$ 615.33, found: 615.9.

General procedure for the synthesis of poly(*n*-butyl acrylate-*co*-MBA) PBA-*co*-MBA$_{xx}$-YY. The example given is for PBA-*co*-MBA$_5$-43.

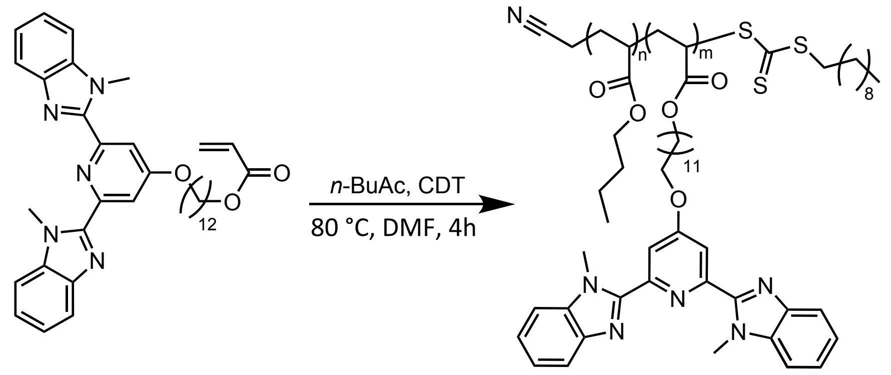

A 50 mL pear-shaped flask was charged with **MBA** (1.96 g, 3.34 mmol), cyanomethyl dodecyl trithiocarbonate (67.8 mg, 0.210 mmol), AIBN (10.80 mg, 0.066 mmol), *n*-butyl acrylate (8.57 g, 9.60 mL, 66.9 mmol), dioxane (500 µL) as an internal reference, and DMF (40 mL) and the mixture was stirred at room temperature until the solid components had dissolved. The reaction mixture was then purged with N_2 for 30 min and stirred vigorously. The reaction was initiated by heating the mixture to 80 °C after which the polymerisation was allowed to proceed for 2 h. The polymerisation was stopped by exposure to atmosphere, cooled to RT and the solution was dialyzed with a membrane with 1000 molecular weight cut-off (MWCO) for 3 days against THF (changed twice day). The solvent was then removed under *vauco* and the resulting solid was dried at 60 °C in *vacuo* overnight. A soft, light red solid was obtained (85% conversion). Different molecular weights were prepared by changing the monomer to cyanomethyl dodecyl trithiocarbonate ratio.

^{1}H NMR (400 MHz, $CDCl_3$): δ 7.93 (s, 2H), 7.86 (s, 2H), 7.45 (s, 2H), 7.36 (s, 4H), 4.24 (s, 8H), 4.03 (s, 42H), 2.27 (s, 20H), 2.00 – 1.20 (m, 165H), 0.93 (s, 61H).

Gel preparation and monitoring

A solution of **PBA-*co*-MBA$_{xx}$-YY** (0.22 g) in $CHCl_3$ (4 mL, run through basic Al_2O_3) was prepared by magnetically stirring. A solution of $Zn(OTf)_2$ (*c*=0.057 M in MeCN) was added in stoichiometric amounts ($[Zn(OTf)_2]$:[Mebip] = 0.5) to ensure complete cross-linking; upon addition, a gel was formed. An additional amount of $CHCl_3$ was added under stirring until a solution had formed. To ensure complete complex formation, 4.5 µL and 9 µL aliquots were taken before and after the addition of the metal salt and additional $CHCl_3$, aliquots were dissolved in 3 mL of solvent (9/1 $CHCl_3$/MeCN v/v) to ensure solubility. These samples were analyzed via UV/Vis spectroscopy (2 mL samples) to confirm complete complex formation. Once this was confirmed, the solvent was evaporated in a well-ventilated fume hood, and the resulting solids were re-swelled in the appropriate solvent (typically chlorobenzene) at the appropriate concentration (typically 12 wt % m/m).

Gel solvent screening

A small-scale version of the general gel preparation was conducted. Aliquots of a given solvent were added until the solvent was no longer absorbed by the gel. The mass of the solvent absorbed was recorded and used to determine the gravimetric swelling capacity of **PBA-*co*-MBA$_{xx}$-YY** for a given solvent.

Preparation of MBTT-loaded gels

The same general procedure was followed for gel preparation but the desired amount of MBTT was added to the **PBA-*co*-MBA$_{xx}$-YY** / metal salt solution before drying.

Photo-induced disassembly of gels

A vial containing the gel was placed on a black sheet of paper and irradiated by three UVA (365 nm) torches from different angles to ensure complete irradiation.

General procedure for UV/Vis titrations

A stock solution of **EH-Mebip** (20 µM) in MeCN was prepared and this concentration was kept constant throughout all titration experiments reported herein. A stock solution of the metal salt in MeCN was prepared from which aliquots were added to the ligand solution. Between the addition of aliquots of the guest solution, a steady state was established by waiting at least one minute before the next addition. Periodically, longer waiting periods were allowed to ensure that no time dependency was at play. UV-Vis absorption spectra were acquired after each addition.

The amount of metal salt required for the complete complexation of the ligands in **PBA-*co*-MBA$_{xx}$-YY** was also established by such titrations. Once this was established, the MSP networks were prepared by adding the entire amount needed for complexation in a single aliquot. For acid decomplexation studies, the procedure was the same as reported above, but in this case, solutions of the complex, formed *in-situ*, were titrated with an acid or TEAC.

Rhodamine B acid titrations

A solution of MBTT in MeCN was prepared (c=9.8 µM) in a quartz cuvette and irradiated with UV light (λ=365 nm, P=1.29 W, power density on the sample ca. 190 mW/cm^2). A solution of Rhodamine B was added to the cuvette (c=1.31 µM). A guest stock solution of dipea (7.18 mM) was prepared in MeCN and titrated in 1-5 µL aliquots. The titration was monitored by UV-Vis absorption spectroscopy.

NMR Characterization

MBP. ^{1}H-NMR spectra (DMSO-d$_6$, 400 MHz) and ^{13}C-NMR (CDCl$_3$, 100 MHz).

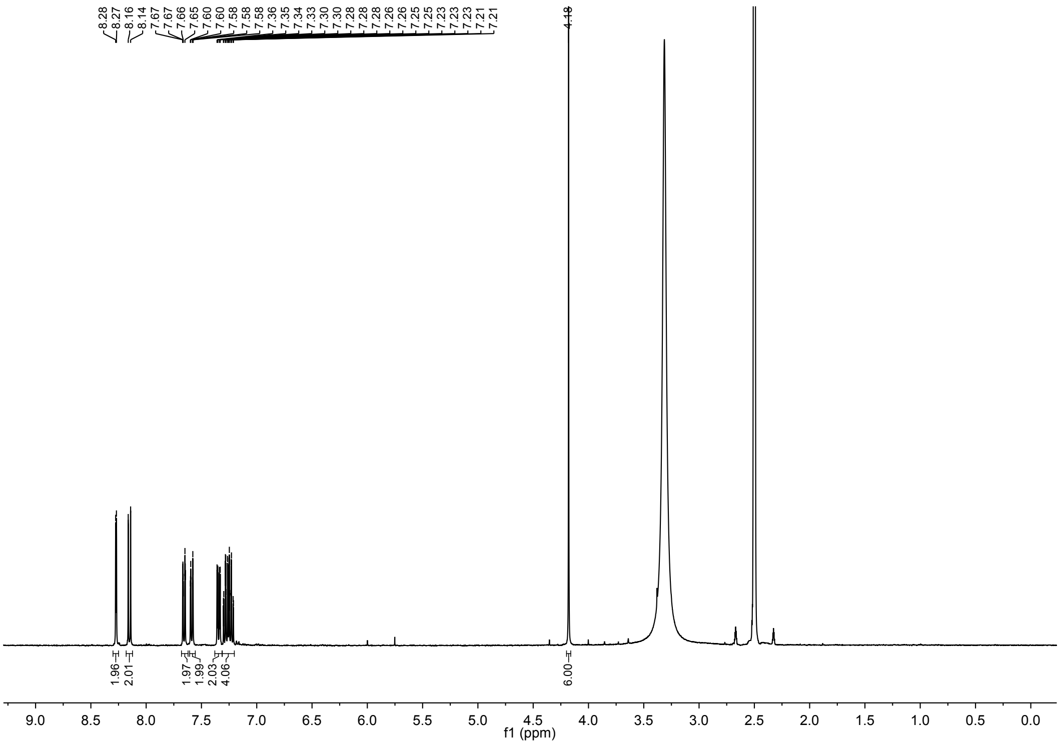

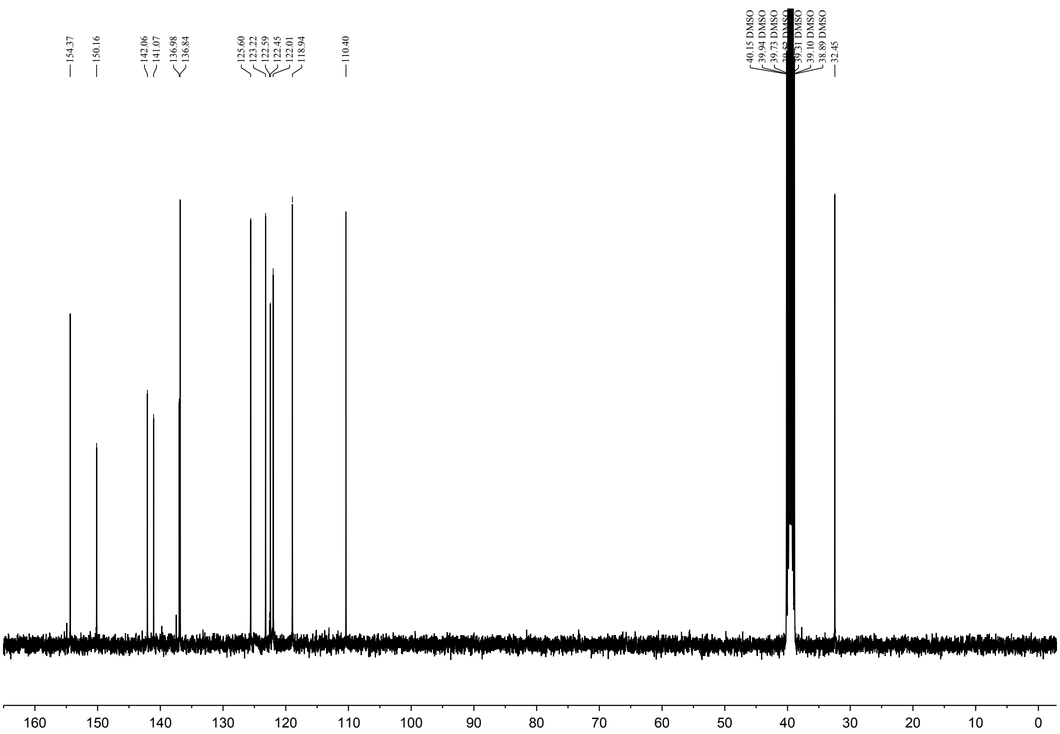

EH-MBP. ^{1}H-NMR spectra ($CDCl_3$, 400 MHz) and ^{13}C-NMR ($CDCl_3$, 100 MHz).

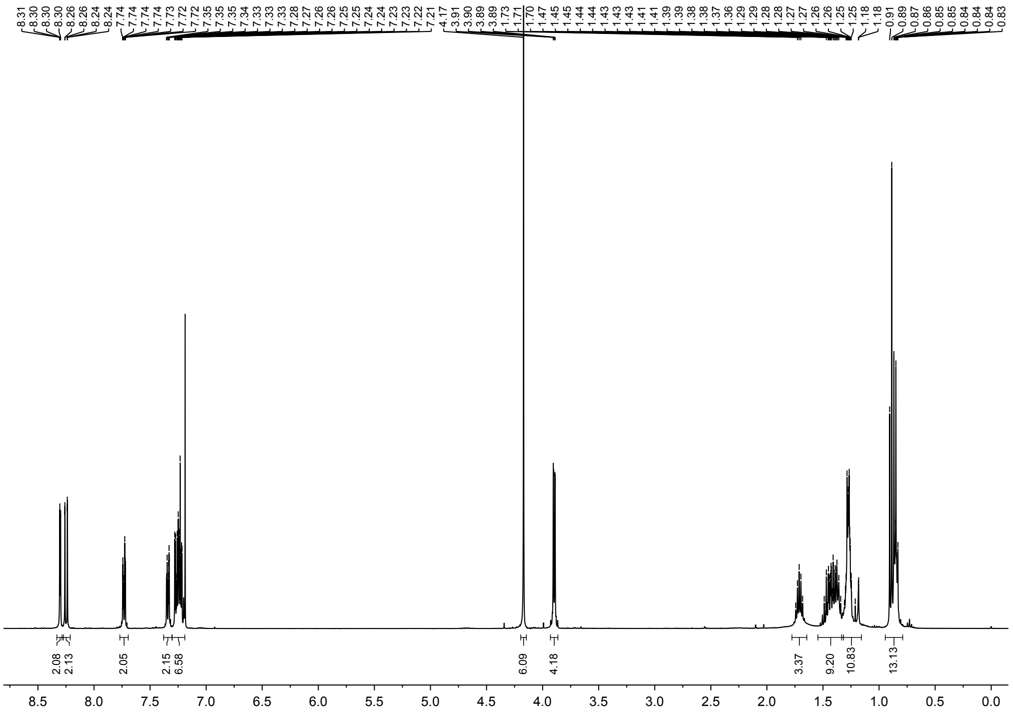

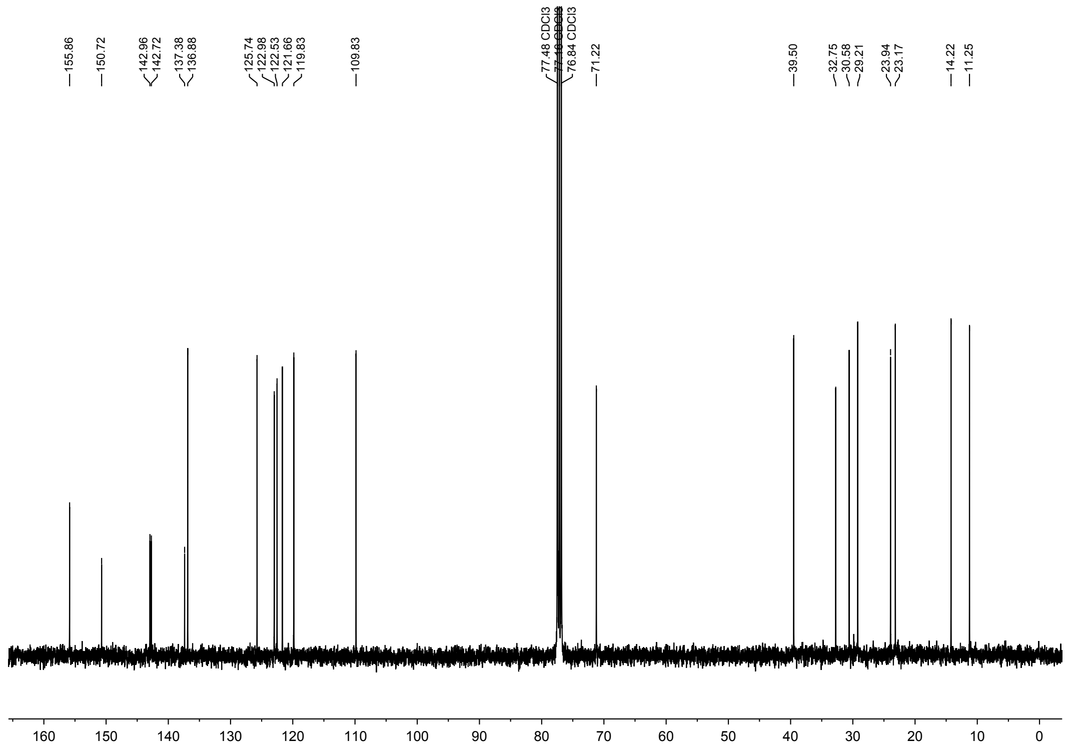

Mebip. ^{1}H-NMR spectra (DMSO-d_6, 400 MHz) and ^{13}C-NMR ($CDCl_3$, 100 MHz).

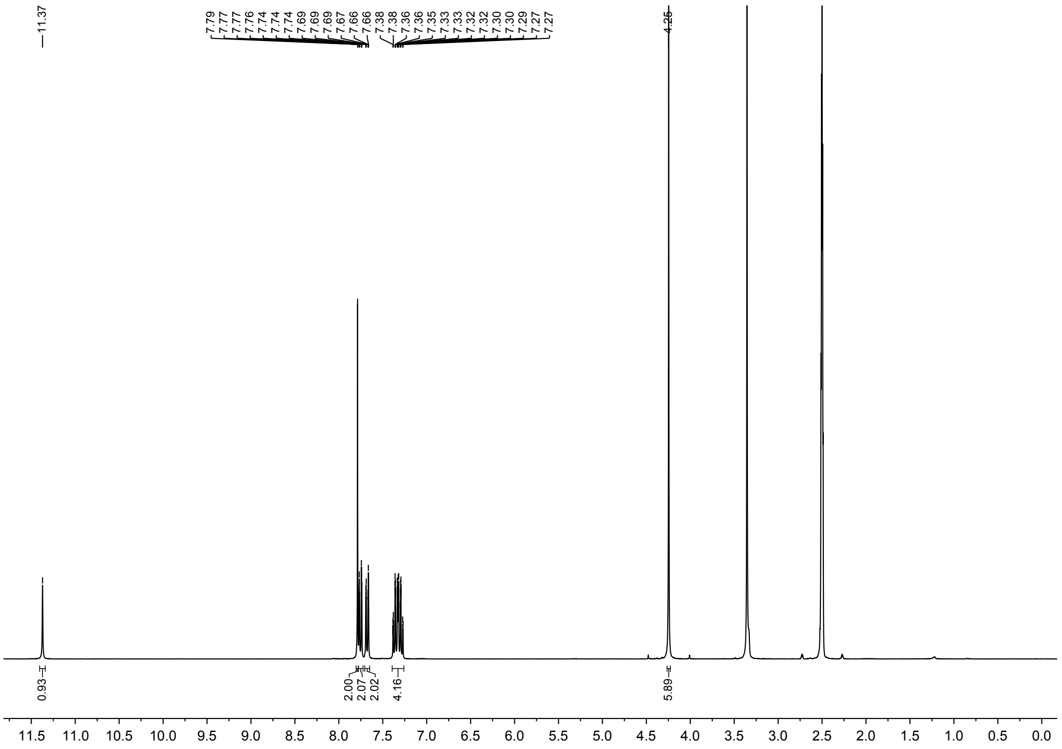

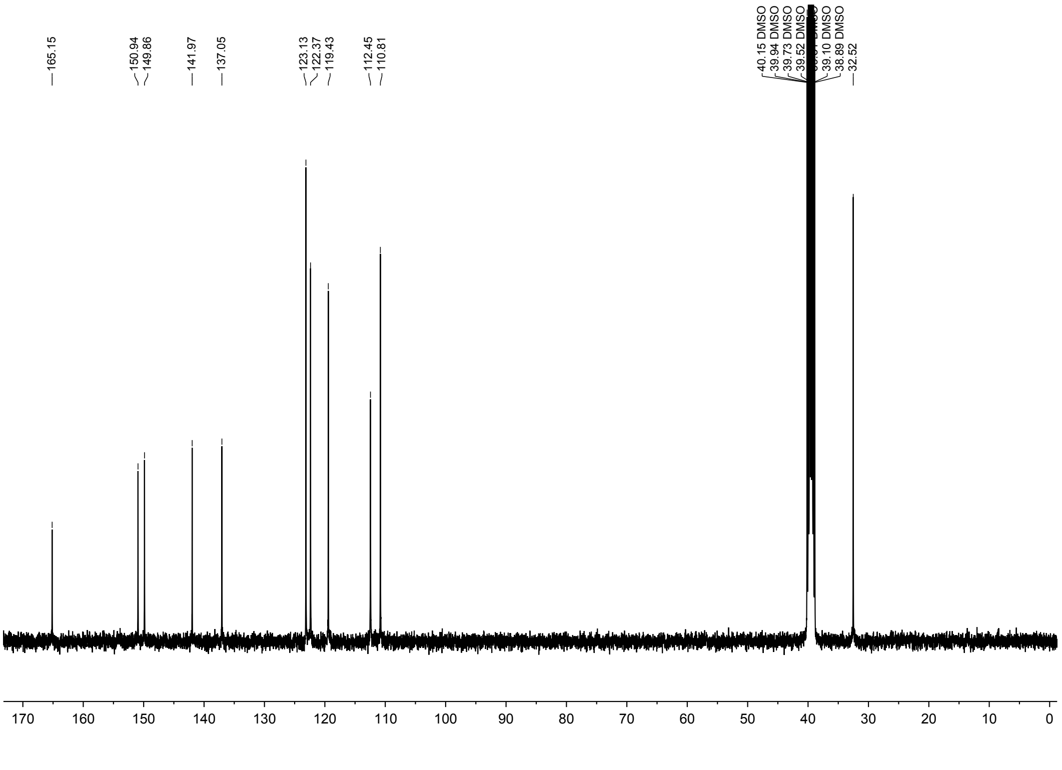

EH-Mebip. ^{1}H-NMR spectra ($CDCl_3$, 400 MHz) and ^{13}C-NMR ($CDCl_3$, 100 MHz).

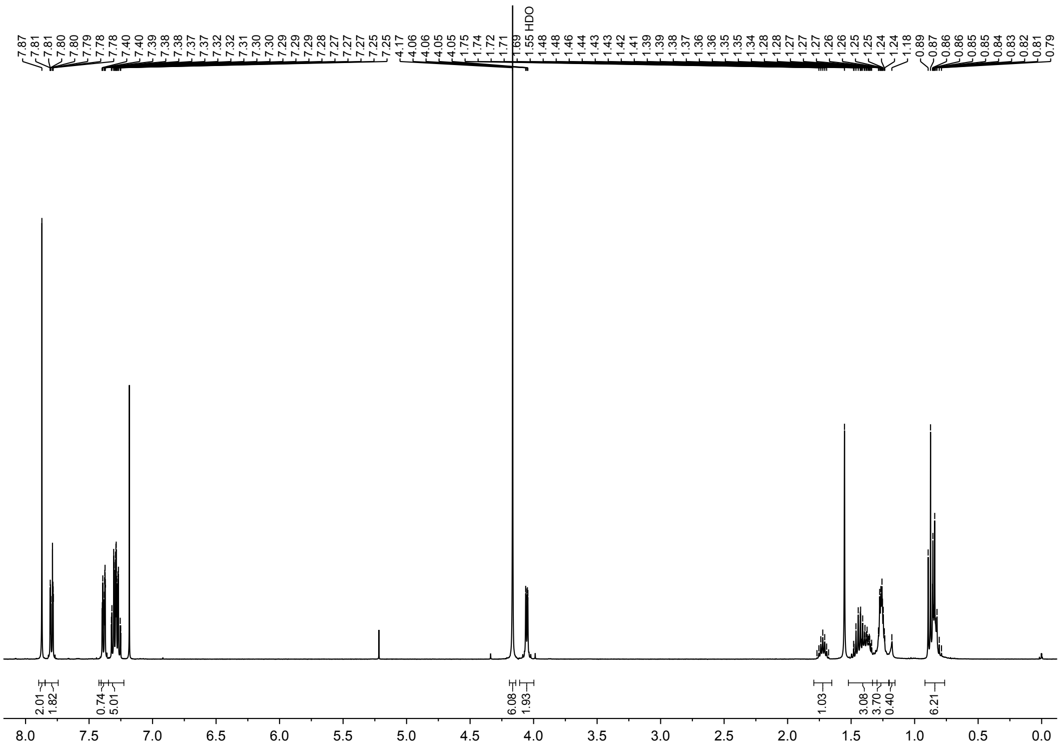

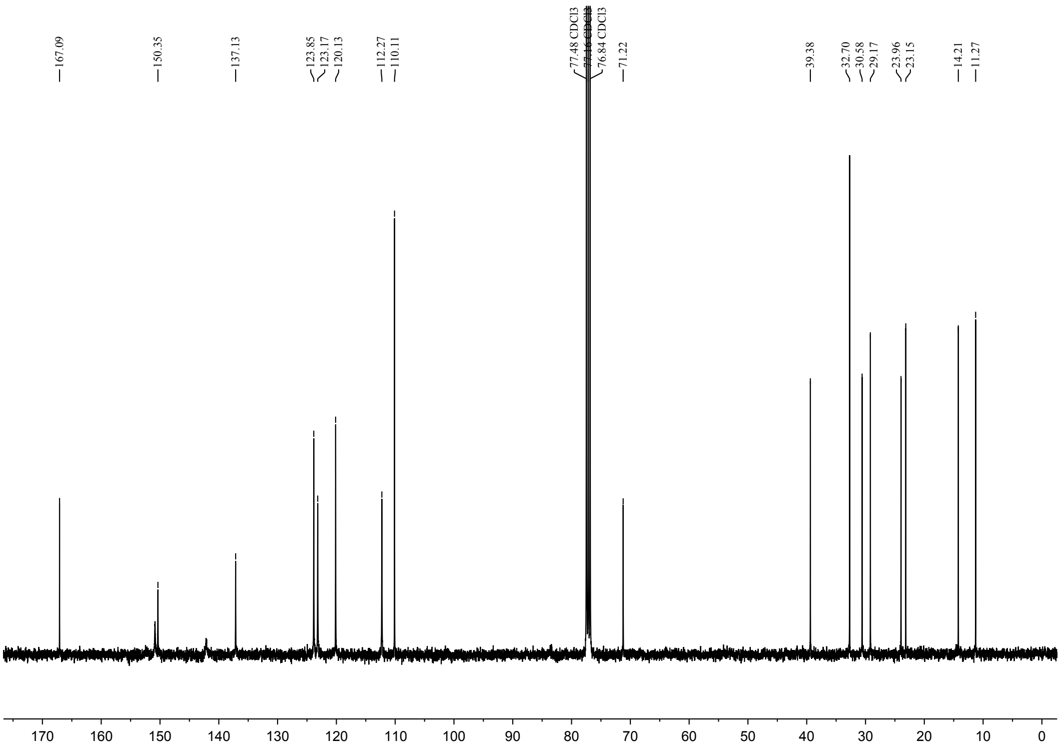

Compound (2). ^{1}H-NMR spectra ($CDCl_3$, 400 MHz) and ^{13}C-NMR ($CDCl_3$, 100 MHz).

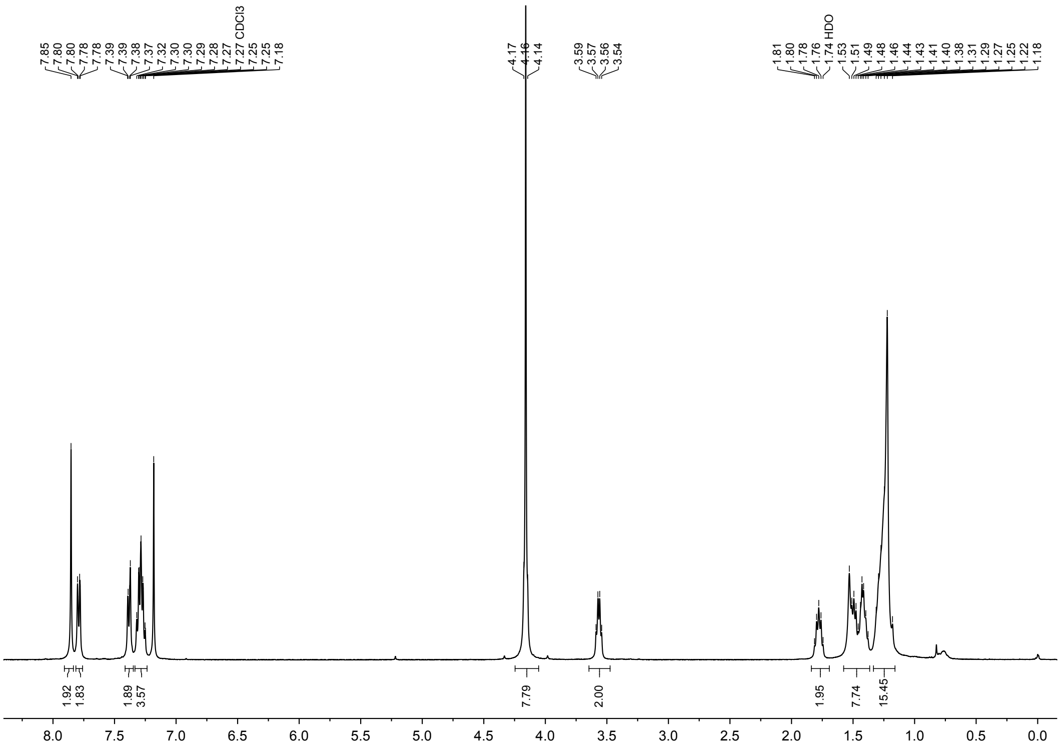

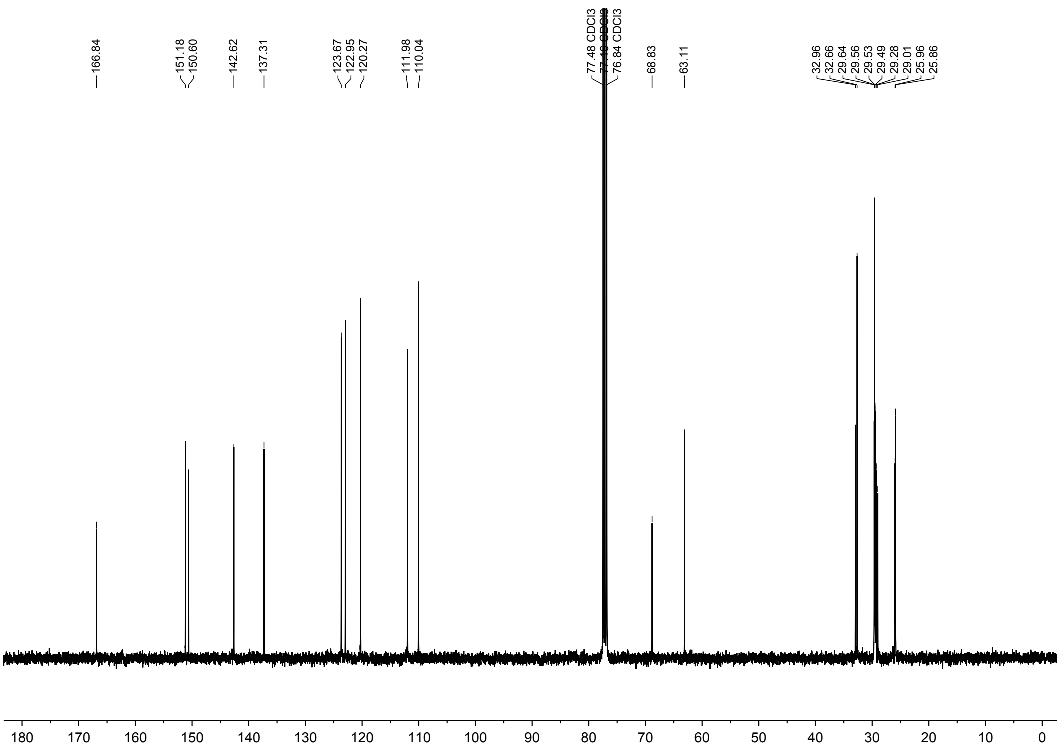

Compound (3, MBA). ^{1}H-NMR spectra ($CDCl_3$, 400 MHz) and ^{13}C-NMR ($CDCl_3$, 100 MHz).

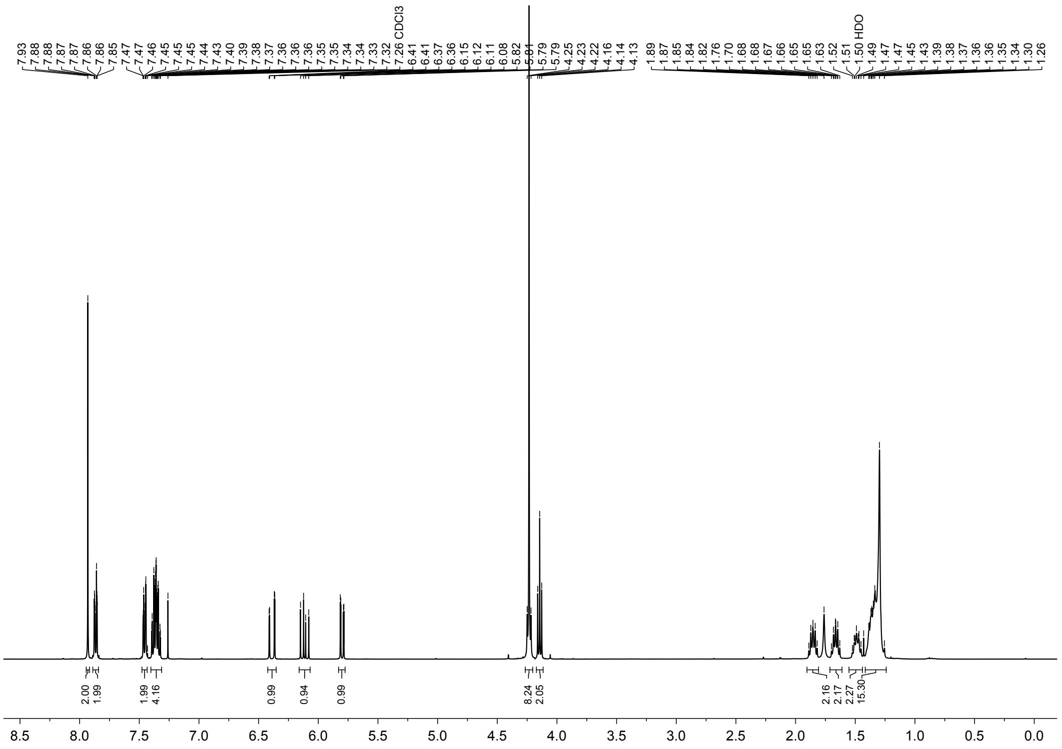

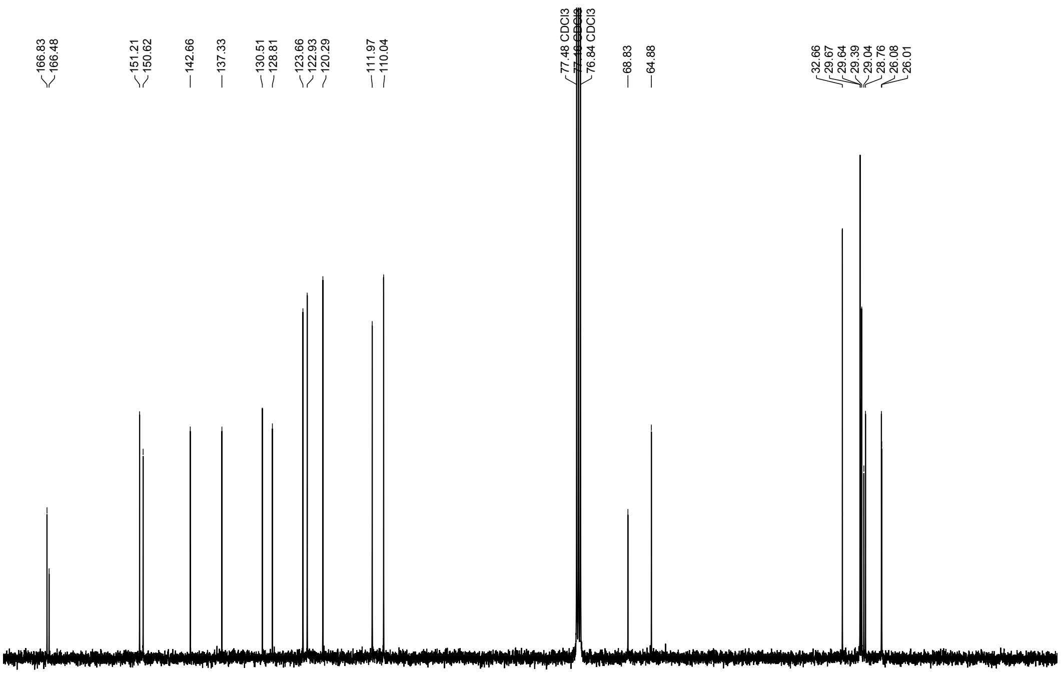

PBA-MBA. ^{1}H-NMR spectra ($CDCl_3$, 400 MHz) and DOSY.

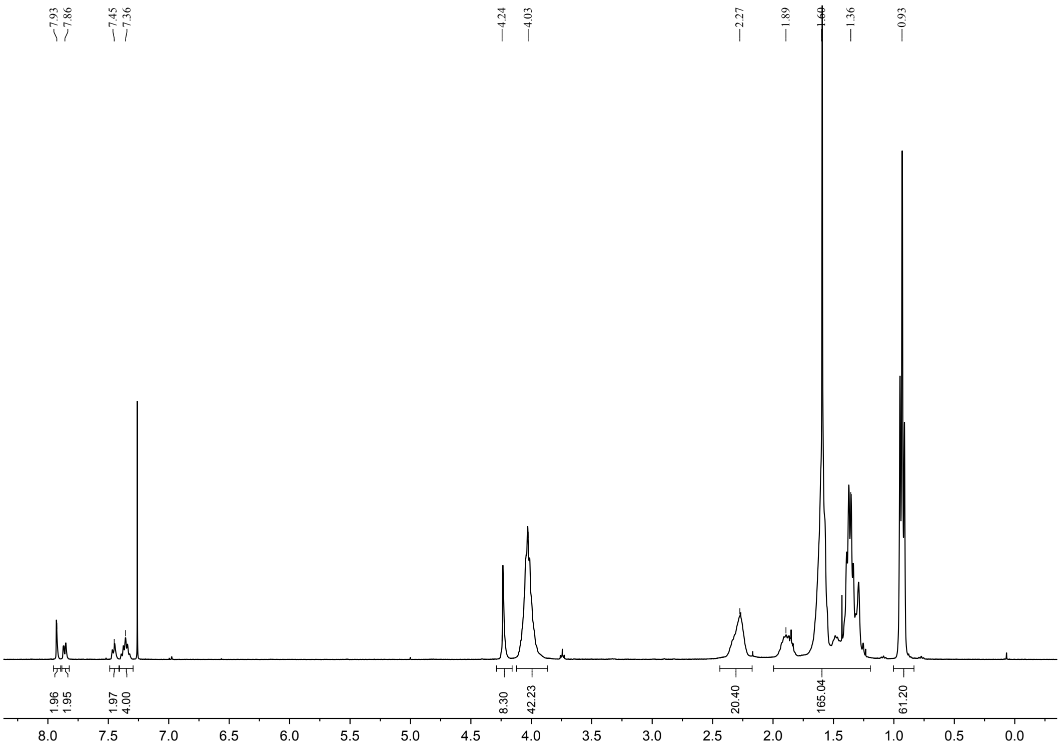

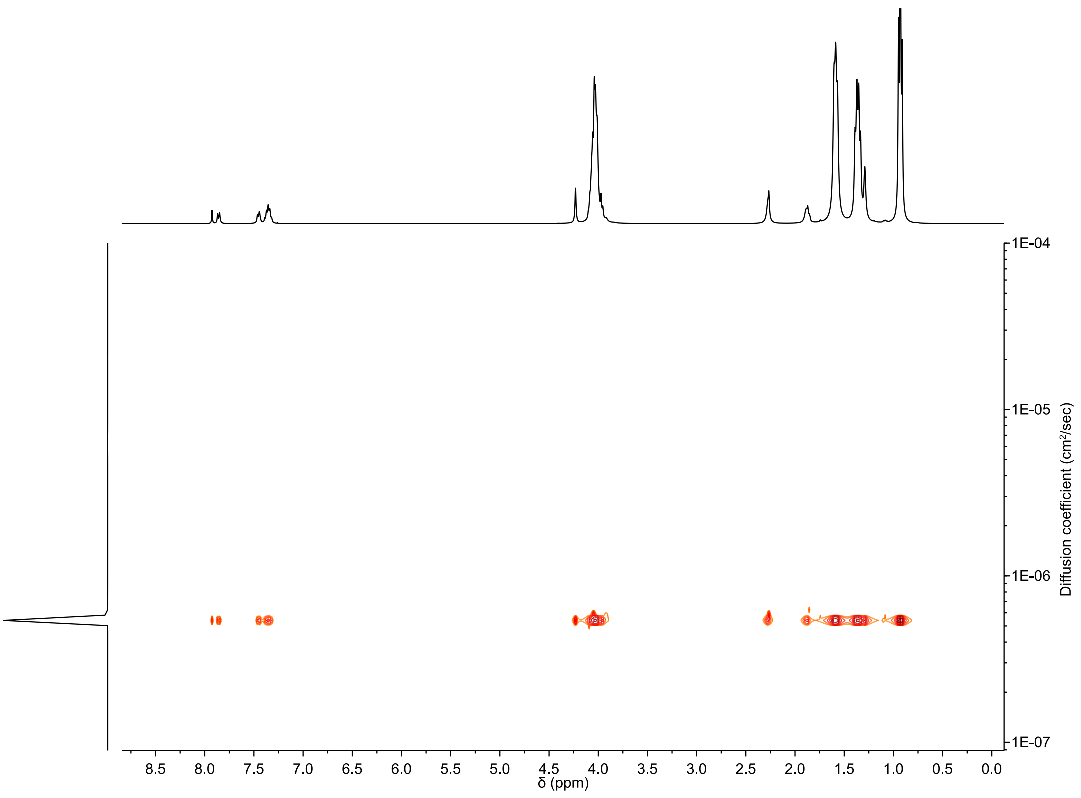

References

1. Beck, J. B.; Ineman, J. M.; Rowan, S. J., Metal/Ligand-Induced Formation of Metallo-Supramolecular Polymers. *Macromolecules* **2005,** *38* (12), 5060-5068.

2. Sautaux, J.; Montero de Espinosa, L.; Balog, S.; Weder, C., Multistimuli, Multiresponsive Fully Supramolecular Orthogonally Bound Polymer Networks. *Macromolecules* **2018,** *51* (15), 5867-5874.
